# Fine tuning deep learning models for breast tumor classification

**DOI:** 10.1038/s41598-024-60245-w

**Published:** 2024-05-10

**Authors:** Abeer Heikal, Amir El-Ghamry, Samir Elmougy, M. Z. Rashad

**Affiliations:** 1 Department of Computer Science, Faculty of Computers and Information, Mansoura University, Mansoura, 35516 Egypt; 2 Department of Computer Science, Misr Higher Institute for Commerce and Computers, Mansoura, 35511 Egypt

**Keywords:** Computer science, Breast cancer, Diagnosis

## Abstract

This paper proposes an approach to enhance the differentiation task between benign and malignant Breast Tumors (BT) using histopathology images from the BreakHis dataset. The main stages involve preprocessing, which encompasses image resizing, data partitioning (training and testing sets), followed by data augmentation techniques. Both feature extraction and classification tasks are employed by a Custom CNN. The experimental results show that the proposed approach using the Custom CNN model exhibits better performance with an accuracy of 84% than applying the same approach using other pretrained models, including MobileNetV3, EfficientNetB0, Vgg16, and ResNet50V2, that present relatively lower accuracies, ranging from 74 to 82%; these four models are used as both feature extractors and classifiers. To increase the accuracy and other performance metrics, Grey Wolf Optimization (GWO), and Modified Gorilla Troops Optimization (MGTO) metaheuristic optimizers are applied to each model separately for hyperparameter tuning. In this case, the experimental results show that the Custom CNN model, refined with MGTO optimization, reaches an exceptional accuracy of 93.13% in just 10 iterations, outperforming the other state-of-the-art methods, and the other four used pretrained models based on the BreakHis dataset.

## Introduction

Breast Cancer (BC) is a leading cause of death worldwide. Annually, the death rates have consistently risen^1^. According to the World Health Organization (WHO), there has been a global increase in BC cases, with around 2.3 million women diagnosed and 685,000 deaths^2^. BC is the most common cancer among women, with 7.8 million new cases reported in the previous 5 years^3^. BTs are of two types: benign and malignant, with the latter having the potential to spread to other parts of the body.

Automated histopathology image classification is one of the most important research fields for examining tissue images, with the aim of improving the decision-making process for diagnosing diseases^4^. The digitization of tissue slides have greatly benefited the ability of pathologists to accurately diagnose diseases, but there are still many morphological and technical variances in the images, making the automatic tissue categorization is a challenging topic^5^.

Histological images of the breast play a crucial role in diagnosing and treating BC, facilitating the classification of histopathological images which is one of the main research fields for studying tissue images. This work focuses on classifying the BreakHis dataset, a commonly utilized collection of BT biopsy images. Hematoxylin and Eosin (H & E) stains are commonly used in histological images for medical diagnosis^6^, allowing pathologists to differentiate between normal and cancerous cells by examining details related to the nucleus’s structure, size, and spatial configuration, as well as the tissue’s composition and density, leading to the grading of the BC^7^.

Deep Convolution Neural Networks (DCNNs) have demonstrated considerable advancements in radiology and imaging sciences such as Alzheimer’s detection^8^ and brain tumor classification^9^, which have impacted the development of Computer-Assisted Diagnostics (CAD)^10^. CAD systems are focused on medical study and are a necessary tool in medical diagnosis^1^. They can automatically categorize histopathological samples into different subvariants of BC, that are urgently needed to aid in accurate diagnosis and decrease the doctors’ workload^11^.

Despite progress in medical science, the diagnosis of BC is still a challenging task in the medical field, with a large number of patients, time-consuming diagnostic stages, and difficulty assessing biopsy outcomes^12^. Against this drawback, this paper aims to classify BTs using Custom CNN and four different pretrained models: MobileNetV3, EfficientNetB0, Vgg16, and ResNet50V2. These are optimized using two different metaheuristic optimizer algorithms to enhance the performance of this work. The two algorithms are (MGTO) and (GWO), where MGTO focuses on global optimization and solving complex engineering problems adapted for hyperparameters fine-tuning tasks due to its ability to efficiently search through large solution spaces, and GWO works in various applications such as parameter tuning, economy dispatch problems, and cost estimating^13^. The optimized parameters are evaluated using different metrics such as accuracy, F1-Score, and ROC-AUC, comparing the results with different related works on the BreakHis dataset. The optimized Custom CNN using MGTO achieved the highest performance compared to other techniques, since it achieves a good balance between exploitation and exploration and also GWO less control over the algorithm because of tuning a few hyperparameters^13^.

The literature reveals a dynamic field of study focused on BC classification, with numerous approaches that demonstrate strategizing the potential of Deep Learning (DL) and image analysis in improving diagnostic outcomes. For instance, research by Rana et al.^14^ on the BreakHis dataset demonstrated a significant accuracy peak for tumor classification, adeptly navigating through imbalanced data sets without initial preprocessing, and they utilized seven different transfer learning models. kolla et al.^15^ presented an improved version of the Swin-Transformer V2 designed specifically for the eight-class classification of BC histopathology images. It uses multi-labeled data and incorporates a sigmoid activation function to effectively manage duplicates and imbalanced data. Additionally, it utilizes focal loss to boost its robustness. These pioneering works, along with others employing quantum computing, approaches and innovative CNN architectures have laid a solid foundation for further exploration. However, a comprehensive review of the literature, particularly focusing on recent studies, indicates specific gaps in the application of advanced CNN models and meta-heuristic optimization techniques in BC classification. Although research has achieved notable successes, there remains a significant opportunity to refine diagnostic accuracy and efficiency through the synergistic use of state-of-the-art machine learning algorithms and optimization strategies. Our study aims to address these gaps by leveraging the latest advancements in CNN architectures and optimization algorithms, thereby providing a novel contribution to the field of BC diagnosis. By comparing our approach with both classical and contemporary methodologies, this paper not only contributes to the ongoing development of BC diagnostic techniques, but also serves as a beacon for future explorations that can further improve and enhance the accuracy of medical diagnostics in oncology. The main contributions of this paper are listed as follows:

- Introducing a distinctive approach for diagnosing BTs using Custom CNN and four other pretrained models.
- Applying two metaheuristic optimizers, GWO and MGTO, to fine-tune the hyperparameters of Custom CNN and the four used pretrained models.
- Exceptional accuracy with MGTO-Custom CNN: Achieving an outstanding accuracy rate of 93.13% within just 10 iterations using the MGTO optimized Custom CNN model represent a significant milestone, surpassing other state-of-the-art techniques.
- Using different metrics like accuracy, F1-Score, loss, (ROC-AUC) curve ensured a comprehensive evaluation of the models.
- Using data augmentation techniques, including random brightness, flips, and rotations, displayed an innovative approach to enhance model robustness and counteract overfitting, which is a common challenge in DL models.

This paper is structured as follows: “Related works” section discusses related works. “Preliminaries” section introduces the basics of metaheuristic optimizers. The proposed work is discussed in “The proposed approach for breast tumor classification” section, followed by an in-depth analysis of its results and a discussion of the findings in “Experimentation and results” section. This paper is concluded in “Conclusion and future works” section with suggested directions for future research.

## Related works

This section discusses various research methods and techniques. Joseph et al.^16^ proposed a BC diagnostic approach using the BreakHis dataset, incorporating data augmentation methods like rotations and shifts. They manually extracted features using the Hu moment, Haralick textures, and color histograms.The accuracy was notable, peaking at 97.87% for $\times$ 40 magnification. The results highlighted the advantages of training at specific magnification levels for enhanced accuracy, emphasizing customized model training’s significance.

In Ref.^17^, a hybrid DLNN method for BC histopathological image classification combined Inception V3’s inception block with ResNet’s residual block. Using the BHI and BreakHis datasets, this approach outperformed traditional Inception, ResNet, and other advanced models recorded accuracies of 0.8655 for BreakHis and 0.8521 for BHI. The importance of data augmentation emerged, emphasizing its role in enhancing dataset diversity, model robustness, and addressing imbalances.

In Ref.^18^, a hybrid classical-quantum neural network approach, trained with transfer learning, was introduced for full-image mammogram classification into malignant and benign categories. The study showcased the method’s ability to generalize complex data, with performance varying among models like AlexNet, VGG19, and others. The approach achieved 84% accuracy compared to contemporary models.

In Ref.^19^, a CNN-based method was introduced for early-stage BC detection using the BreakHis 400x magnification from Kaggle. Among the architectures, including NASNet-Large, DenseNet-201, and Inception ResNet-V3, the Big Transfer (M-r101x1x1) achieved the top accuracy at 90%. The primary focus was the precise identification of BC using the selected Neural Networks (NNs).

Karthik et al.^20^ introduced an ensemble of two custom DCNNs, CSAResnet and DAMCNN, leveraging Channel and spatial attention mechanisms for histopathology image features. Enhanced by ensemble learning, this framework achieved 99.55% accuracy on the BreakHis dataset. The high accuracy is attributed to the synergistic combination of two NNs, attention mechanisms, data augmentation, the Resnet-101 foundation, and advanced optimization. This comprehensive strategy ensured optimal detection outcomes.

Umer et al.^21^ presented a DL method for multi-class BC classification using a 6B-Net deep CNN model enhanced with feature fusion and selection. Evaluated on BreaKHis with 7909 images across eight classes and another BC histopathology dataset of 3771 images in four classes, the approach achieved 94.20% accuracy for the four-class set in 226 s and 90.10% for the eight-class set in 147 s.

Aljuaid et al.^22^ presented a computer-aided diagnostic approach for BC classification using a fusion of DNNs (ResNet 18, ShuffleNet, and Inception-V3Net) and TL on the BreakHis dataset. Binary classifications achieved accuracies of 99.7%, 97.66%, and 96.94% for ResNet, InceptionV3Net, and ShuffleNet, respectively. For multi-class classifications, accuracies were 97.81%, 96.07%, and 95.79%.

Duzyel et al.^23^ introduced an “adaptive resizer” using CNN architecture to optimally resize high-resolution histopathological images for BC diagnosis. When integrated with DL models, notably DenseNet201, and tested on the BreakHis dataset, the approach achieved 98.96% accuracy for $\times$ 40 magnified images, outperforming traditional resizing methods like bilinear interpolation.

Kumari et al.^24^ employed transfer learning AI like VGG-16, Xception, and Densenet-201, for BC classification from histopathological images. Using the IDC and BreaKHis datasets and data preprocessing methods, the system achieved accuracies of 99.42% and 99.12%, respectively. The model’s efficacy remained consistent across varying image magnifications. While fine-tuning details were shared, results before and after weren’t explicitly stated.

Rana et al.^14^ used the BreakHis dataset to automate tumor classification, efficiently handling imbalanced data without preprocessing. By employing seven transfer learning models and resizing images to 224 $\times$ 224 pixels, the study found that the Xception model had the highest accuracy of 83.07%. Notably, DarkNet53 showed the best balance accuracy of 87.17% for imbalanced data. This research provides guidance for medical professionals in choosing suitable models for tumor classification in imbalanced datasets.

Ijaz et al.^25^ introduced the CBAM-VGGNet model, a fusion of VGG16 and VGG19, specifically trained on cancerous histopathology datasets. The model’s complexity was streamlined using the GAP layer and CBAM, ensuring a focus on vital features. Additionally, a hybrid pre-processing technique enhanced image clarity. On testing with the BreakHis dataset, the model boasted 98.96% accuracy and a 97.95% F1-Score, surpassing numerous top-tier models.

Ali et al.^26^ enhanced BC classification precision by merging meta-learning with CNNs. Using the BUSI dataset to categorize breast lesions, they overcame traditional method challenges with models like Inception V3, ResNet50, and DenseNet121, combined with preprocessing. Their ensemble model reached 90% accuracy, emphasizing meta-learning’s potential in medical imaging.

Maleki et al.^27^ enhanced histopathological image classification using transfer learning and six pre-trained models, classifying with XGBoost. Using the BreakHis dataset, the DenseNet201 and XGBoost combination achieved 91.925% accuracy. The research emphasized layer-specific fine-tuning in AlexNet and the significance of magnification. Chakravarthy et al.^28^ integrated DL with metaheuristic algorithms to enhance BC severity classification. Utilizing MIAS, INbreast, and WDBC datasets, their approach transformed non-linear features using optimization’s techniques. Compared to Gaussian Naïve Bayes and LSVM, the CSO-wKNN method showed higher accuracies of 84.35%, 83.19%, and 97.36% across the datasets, respectively.

Sharma et al.^29^ applied transfer learning to histopathological image classification using the BreakHis dataset. By optimizing AlexNet at specific depths for various magnifications, they achieved accuracies of 89.31% for 40$\times$, 85.75% for 100$\times$, 83.95% for 200$\times$, and 84.33% for 400$\times$, emphasizing the importance of tailored approaches in this domain.

Iqbal et al.^30^ introduced an Adaptive Hyperparameter Tuning (AHT) algorithm for enhancing CNN-based medical image classification. Their method achieved 91.08% accuracy on the BraTS dataset and 91.26% on the BreaKHis and 93.21% on the NIH X-ray.

Maan et al.^31^ introduced a saliency detection system for BC using DL. Utilizing the BreakHis dataset, they applied VGG16 and ResNet to detect and classify cancer regions across five diagnostic categories, achieving 96.7% training accuracy and 90.4% testing accuracy.

Hirra et al.^32^ introduced a BC classification method on histopathology images using the Deep Belief Network (DBN) paired with logistic regression. The network autonomously extracts image patch features, which are then classified via logistic regression, achieving an accuracy of 86%.

Saxena et al.^33^ developed ten BC diagnosis models using the BreakHis dataset with pre-trained CNNs and an SVM classifier. ResNet50, ResNet101, and AlexNet were highlighted as top feature extractors for histopathology images. Model performance varied by magnification, with the AlexNet-SVM model excelling, achieving 89.46% and 89.78% accuracy at 40$\times$ and 100$\times$ magnifications, respectively.

Gour et al.^34^ introduced a breast histopathological image classification method using ResNet, a 152-layered convolution network. Extracting rich features from the images, it achieved 92.52% accuracy and a 93.45% F1-Score when used with an SVM classifier, on the BreakHis dataset, the method outperformed contemporary techniques for differentiating benign from malignant images. Table 1 illustrates more details about some of the related works.

**Table 1:** Summary of some related works.

Study	Year	Dataset	Preprocessing	Augmentation	Feature extractor	Classification technique	Results
Joseph et al.^16^	2022	BreakHis	–	Rotation (90^∘^ and 180^∘^) width shift (0.2%), height shift (0.2%), shear range horizontal (0.2%), horizontal flip, fill mode (constant), range scale (true), noise disturbance, geometric transformation.	Handcrafted techniques: Hu moment (for shape extraction), Haralick textures (for texture extraction), Color histogram (for color extraction).	Deep Neural Network (DNN) with four dense layers and Softmax as activation for the last layer. Dropouts and Adam optimization algorithm are also used in the DNN.	Acc: 97.87% for $\times$ 40 magnification 97.60% for $\times$ 100 magnification 96.10% for $\times$ 200 magnification 96.84% for $\times$ 400 magnification.
Singh et al.^17^	2022	Breast histopathology images (BHI) BreakHis	–	–	Inception block of Inception V3, Residual block of Resnet.	Hybrid DNN (comprising of inception and residual blocks)	Acc: 80.80% for BreakHis dataset (40$\times$), 82.76% (100$\times$), 86.55% (200$\times$), and 85.80% (400$\times$), 85.21% for BHI dataset.
Karthik et al.^20^	2022	BreakHis	Image processing: resized from 700 $\times$ 460 pixels to 224 $\times$ 224 pixels.	Image Data Generator	**1.**CSAResnet (Channel and Spatial Attention embedded Resnet-101): Utilizes a pre-trained Resnet-101 model, and Integrates channel and spatial attention modules for better feature refinement. **2. **Dual Attention Multiscale Convolutional Neural Network (DAMCNN): Combines Densenet-201 (with channel and spatial attention) and Efficientnet-B0, and employs global average pooling on both architectures to generate feature vectors which are then fused.	Weighted ensemble learning, where contributions of both the CSAResnet and DAMCNN models are weighted by their individual performances.	Acc: 99.55%, Precision: 99.44%, Recall: 99.71%, F1-Score: 0.996.
Umer et al.^21^	2022	BreakHis	–	–	Uses a 35-layer deep CNN model for feature extraction. Feature vectors are passed as input to the PSO feature selection algorithm for the best feature selection.	Deep 6B-Net with deep feature fusion and selection technique	Average accuracy: 94.20 % for four classes, and 90.10 % for eight classes.
Aljuaid et al.^22^	2022	BreakHis	Image editing to remove noise, undesired traces, and variations in brightness or color. Used filters: Median filter and Gaussian filter. Resized to 224 $\times$ 224 for ResNet18 and ShuffleNet, and 299 $\times$ 299 for Inception-V3Net.	Techniques: Random reflection, multiple rotations, and translations.	ResNet 18, Inception-V3Net, and ShuffleNet.	DNNs: ResNet 18, ShuffleNet, Inception-V3Net (pre-trained on the ImageNet database).	Acc: 99.7% for ResNet binary class, and 97.81% for multi class, Inception-V3Net: 97.66% for binary class, and 96.07% for multi class, ShuffleNet: 96.94% for binary class, and 95.79% for multi class.
Maan et al.^31^	2022	BreakHis	–	Image Data Generator. Parameters for augmented images include zoom range, shear range, rotation range, width shift range, height shift range, horizontal flip, and fill mode.	CNN method with architectures VGG16 and ResNet.	DCNN based methodology using convolutional network layers and activation functions. Neurons generate linear output with convolution as a primary operation.	Training accuracy: 96.7%, Testing accuracy: 90.4%.
Duzyel et al.^23^	2023	BreakHis	Resizing with a learnable adaptive resizer (448 × 448). Bilinear rescaling.	Classical augmentation techniques from the Keras libraries, such as zooming and vertical-horizontal flips, were utilized. By incorporating these augmented data into the training set.	Fully connected network consisting of multiple layers for binary classification.	Extracting features using various CNN models: VGG16, VGG19, MobileNetV2, InceptionResnetV2, DenseNet121, DenseNet201, and EfficientNetB0.	TL involving feature-based and fine-tuning transfer learning. With adaptive resizer: The VGG19 model trained with the adaptive resizer was able to learn faster in the first 20 epochs and showed more stability during training. DenseNet201 achieved the highest accuracy 98.96% for 40$\times$
Kumari et al.^24^	2023	IDC, BreakHis	Resize (224 × 224 × 3), Flips, Zoom	Transfer Learning	VGG-16, Xception, Densenet-201.	Proposed Target.	Acc: 99.42% for IDC, and 99.12% for BreakHis.
Rana et al.^14^	2023	BreakHis	Resize	–	Transfer Learning.	Seven Models.	Acc of Xception with unbalanced dataset: 83.07%
Ijaz et al.^25^	2023	BreakHis	Median filter, CLAHE	Contrast Enhancement, Zoom	VGG16, VGG19.	VGG16+VGG19.	Acc: 94.44% (40$\times$), and 97.61% (100$\times$).
Ali et al.^26^	2023	BUSI	Resize to (300 × 300) dimensions, Normalization	–	Inception, ResNet, DenseNet.	Logistic Regression.	Acc: 90% .
Maleki et al.^27^	2023	BreakHis	Image resizing (from 700$\times$460 to 227$\times$227)	Rotation, flipping, zooming, and random rotation.	Pretrained models (specifically mentioned: DenseNet201 and AlexNet).	Extreme Gradient Boosting. (XGBoost).	Acc: 93.6 % (40$\times$), 91.3% (100$\times$), 93.8 % (200$\times$), and 89.1 % (400$\times$). The average accuracy = 91.925 %.
Hirra et al.^32^	2021	Various datasets including histopathology images from the four different data cohorts, Hospital of the University of Pennsylvania (HUP), Case Western Reserve University (CWRU), Cancer Institute of New Jersey (CINJ), and The Cancer Genome Atlas (TCGA).	Crop, Grayscale, Gaussian	–	Deep Belief Network.	Backpropagation NN.	Acc: 86.00%.
Saxena et al.^33^	2020	BreakHis	Resize, Crop, Patch	–	Pretrained CNNs.	Linear SVM.	Acc: 88.00%.
Gour et al.^34^	2020	BreakHis	Histopathological image resizing (specified sizes for different models like 227 $\times$ 227 $\times$ 3 for AlexNet, 299 $\times$ 299 $\times$ 3 for Inception-v3, and 224 $\times$ 224 $\times$ 3 for others) Transfer learning (where last three layers are replaced and fine-tuned).	Stain normalization Image patches generation Affine transformation (including image rotation and flipping).	CNNs(specifically ResHist model) Residual learning.	ResHist model (for direct classification) KNN, RF, QDA, SVM (using deep features extracted from ResHist).	Without data augmentation accuracy is 84.34% for ResHist model F1-Score = 90.49%. With data augmentation, accuracy is 92.52% and F1-Score is 93.45%.

## Preliminaries

This section aims to provide a foundational understanding of two metaheuristic algorithms, namely Grey Wolf Optimization (GWO) and Modified Gorilla Troops Optimization (MGTO).

### Metaheuristic algorithms

Meta-heuristic algorithms, unlike conventional ones, use a random approach in their search space, allowing them to cover a larger portion more efficiently^35^. They use search information from each iteration to guide subsequent searches and have a reduced risk of getting trapped in local optima^36^. Two such algorithms, GWO and MGTO are employed to determine optimal values for the hyperparameters of the Custom CNN and the four pretrained models, including Mobile-NetV3, EfficientNetB0, Vgg16, and ResNet50V2.

####  Grey wolf optimization (GWO)

GWO mimics grey wolf behaviors^37^. It uses a hierarchical structure within a wolf pack, with the alpha wolf being the best solution found, and beta and delta wolves represent the second and third best solutions, respectively. This method ensures that the optimization process leans towards the top three solutions. The primary equations driving this method include^38^, Equations (1) and (2) represent the encircling behavior of the grey wolves:

1 $$\begin{aligned} D= & {} |C.XP(t) - X(t) |, \end{aligned}$$

2 $$\begin{aligned} X(t + 1)= & {} XP(t) - A.D, \end{aligned}$$

where $D$ represents the distance between the current position $X(t)$ of the grey wolf and the position of the prey $XP(t)$. $A$ and $C$ are coefficient vectors. $X(t)$ is the position vector of a grey wolf at time $t$. $XP(t)$ is the position vector of the prey at time $t$. The coefficient vectors are calculated in Eqs. (3) and (4):

3 $$\begin{aligned} A= & {} 2a \cdot r1 - a, \end{aligned}$$

4 $$\begin{aligned} C= & {} 2 \cdot r2, \end{aligned}$$

where $r1$ and $r2$ are random vectors in the range [0, 1]. The components of a vector $a$ are linearly decreased from 2 to 0 over the course of iterations. Therefore, the hunting process involves searching for prey (exploration), encircling the prey, and finally attacking the prey (exploitation). This process is guided by the alpha, beta, and delta wolves, which represent the top three solutions in terms of fitness value. All other candidate solutions (omega wolves) update their positions with respect to these three best search agents.

GWO’s advantages are that it efficiently solves single and multiobjective problems because of its good local search criteria, performing exceptionally well across different problem types and solutions. One of the drawbacks of GWO is that having fewer parameters to fine-tune could lead to a decrease in algorithm control. Additionally, when dealing with single-peaked (unimodal) problems, the algorithm’s speed may decrease towards the end of the process as it helps to find the optimal solution, despite initially expediting the process^13^.

#### Modified Gorilla Troops Optimization (MGTO)

The Gorilla Troop Optimization (GTO) algorithm is a metaheuristic optimization method that mimics gorilla social behavior^39^. Modified Gorilla Troops Optimization (MGTO) provides three strategies that take into account the insufficient convergence accuracy of the GTO algorithm and its low convergence speed^40^.

The first MGTO strategy introduces a shrinkage control factor fusion to expand the search space and reduce search blindness by strengthening the communication between silverback gorillas and other gorillas to improve global optimization performance. The second strategy introduces a sine-cosine interaction fusion based on closeness to stabilize the performance of silverback gorillas and other gorilla individuals and improve the convergence ability and speed of the algorithm. Finally, the third strategy of MGTO introduces gorilla individual difference identification to reduce the difference between gorilla and silverback gorillas to improve the quality of the optimal solution.

During the initialization phase, the MGTO algorithm initializes the population $X_i$ by generating it randomly and sets the initial position of the silverback gorilla. As the exploration phase begins, the algorithm implements the original algorithm’s three mechanisms: updating individual positions through comparison and introducing a fusion strategy for shrinkage control factors. This is the first strategy that expands the search space of the algorithm and decreases search blindness. it expressed as follows:

5 $$\begin{aligned} CAN = e^{1- {\frac{t}{Max\_It}}} \times \cos \left( \frac{t}{2} + \frac{\pi }{4}\right) , \end{aligned}$$

where *CAN* represents a contraction factor which enables gorillas to explore more unknown spaces based on the experience level *U* of the gorillas, *t* represents the current iteration, $Max\_It$ is the maximum number of iterations, and *e* is the base of the natural logarithm. Equation (6) calculates the experience level of the gorillas based on fitness scores.

6 $$\begin{aligned} U = \frac{Fi - Silverback\_Score}{Mean - Silverback\_Score}, \end{aligned}$$

where *Fi* refers to the fitness value of the $i^{th}$ gorilla, $Silverback\_Score$ represents the fitness value of the silverback gorilla, and $Mean$ refers to the average fitness value of all gorillas. The updated Equations When $U > 1$ are expressed as follows:

7 $$\begin{aligned} GX_i= & {} \left[ \frac{(UB - LB) \times (|CAN| - rand) \times \text {rand}(1, \text {dim})}{2}\right] + LB, |CAN| \ge 0.5, \end{aligned}$$

8 $$\begin{aligned} GX_i= & {} (X_i - X_{r1}) \times D, |CAN| < 0.5, \end{aligned}$$

where *UB* and *LB* refer to the upper and lower bounds, rand refers to a random number between 0 and 1, rand(1,dim) refers to a random vector with a problem dimension ranging from 0 to 1 with a uniform distribution, dim represents the dimension of the problem, $X_{r1}$ represents a random gorilla individual, and D refers to a random vector with problem dimension generated in the interval |*CAN*| with uniform distribution.

In the exploitation stage, the algorithm follows the silverback gorilla and competes for adult female gorillas. It integrates the sine cosine interaction fusion strategy, which is based on closeness to enhance the algorithm’s convergence ability and speed while stabilizing the performance of individual silverback gorillas and other gorillas. Finally, it enhance the quality of the optimal solution by using gorilla individual difference identification strategy that introduced by MGTO to decrease the difference between gorillas and silverback gorillas. The equations of exploitation stage are detailed in Ref.^40^.

MGTO can potentially be adapted for hyperparameter optimization tasks due to its ability to efficiently search through large solution spaces. The goal is to find the optimal set of hyperparameters for a given model.

## The proposed approach for breast tumor classification

**Figure 1:**
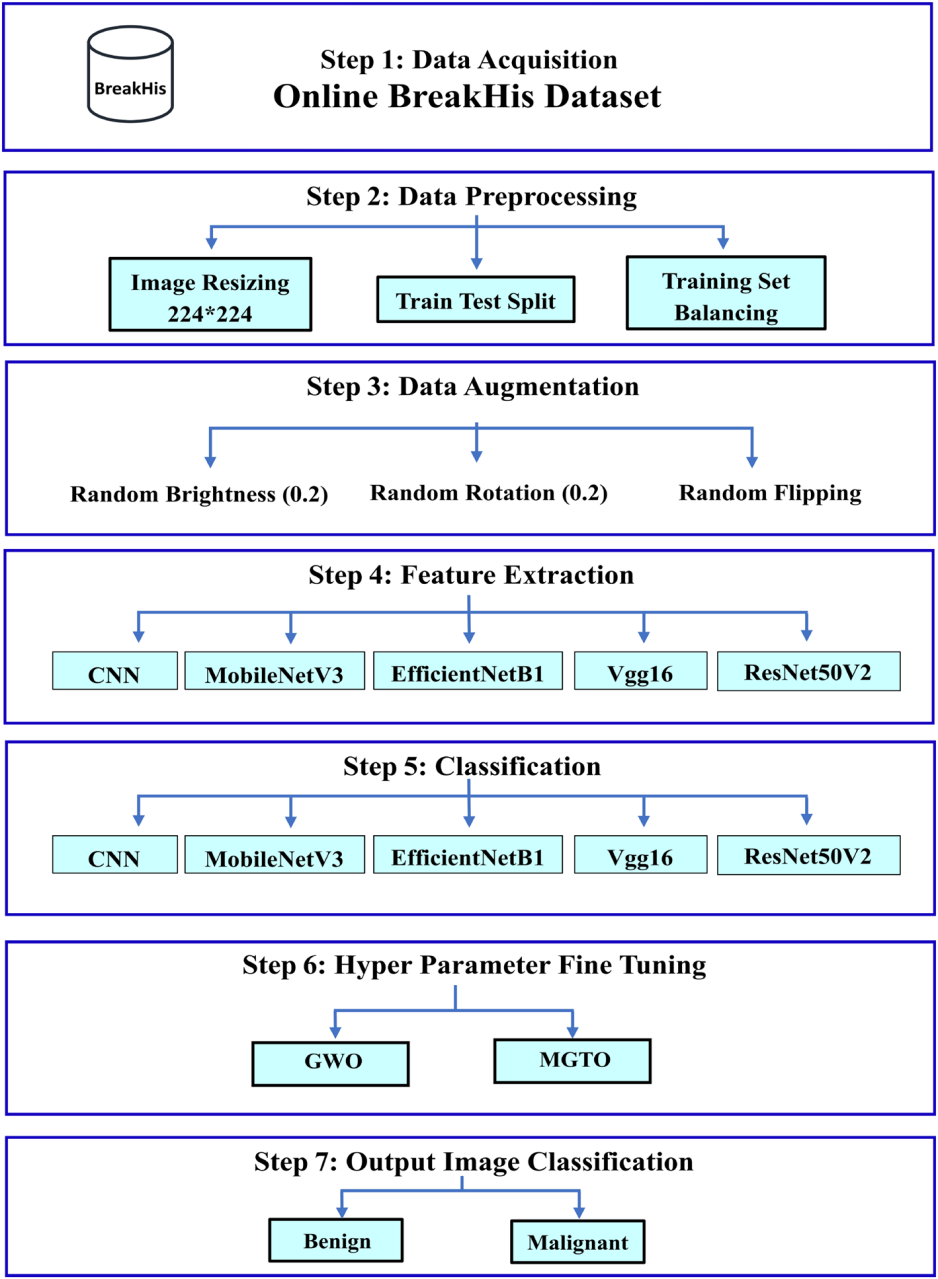
Stages of the proposed approach.

This paper proposes a diagnostic approach utilizing computer vision models to differentiate between benign and malignant BTs using histopathology images from the BreakHis dataset. As shown in Fig. 1, the main stages involve preprocessing, which encompasses image resizing, data partitioning (training and testing sets) and training set balancing, followed by the data augmentation technique. Both feature extraction and classification tasks are employed by a Custom CNN and four pretrained models: MobileNetV3, EfficientNetB0, Vgg16, and ResNet50V2. Hyper-parameter tuning using both GWO and MGTO optimizers is applied. The performance is evaluated using different metrics with detailed explanations in subsequent sections.

### Data acquisition

The BreakHis dataset, sourced from Kaggle, features 7909 histopathological images of BT tissues obtained from 82 patients^41^. Table 2 presents the number of images at each magnification. These images are saved as PNGs have a 700x460 pixel resolution and exhibit a 3-channel RGB color scheme. The dataset includes 2,480 benign and 5,429 malignant samples, with each channel carrying an 8-bit depth.

**Table 2:** BreakHis dataset structure.

Tumor type	Magnification				Total
	$\times$ 40	$\times$ 100	$\times$ 200	$\times$ 400	
Benign	652	644	623	588	2480
Malignent	1370	1437	1390	1232	5429

### Data preprocessing

Data preprocessing significantly influences the efficiency of DL algorithms. It involves image resizing, balancing training set, and the formatting of data in a manner that facilitates the algorithm’s comprehension. The current method adopts the subsequent preprocessing techniques.

#### Image resizing

To ensure compatibility with pretrained models and commonly used algorithms for image classification tasks, image resizing is performed as a standard preprocessing step. Resizing the images to a specific size (224 $\times$ 224) pixels. The choice of resizing the images to a standard size, specifically 224 $\times$ 224 pixels, was motivated by the compatibility requirements of the pretrained models employed in our analysis. Many pretrained models including MobileNetV3, EfficientNetB0, Vgg16, and ResNet50V2, are trained on datasets where images are commonly resized to this standard size, which is a common practice in computer vision tasks as it enables fair comparisons between different models and methodologies.

#### Train test split

**Figure 2:**
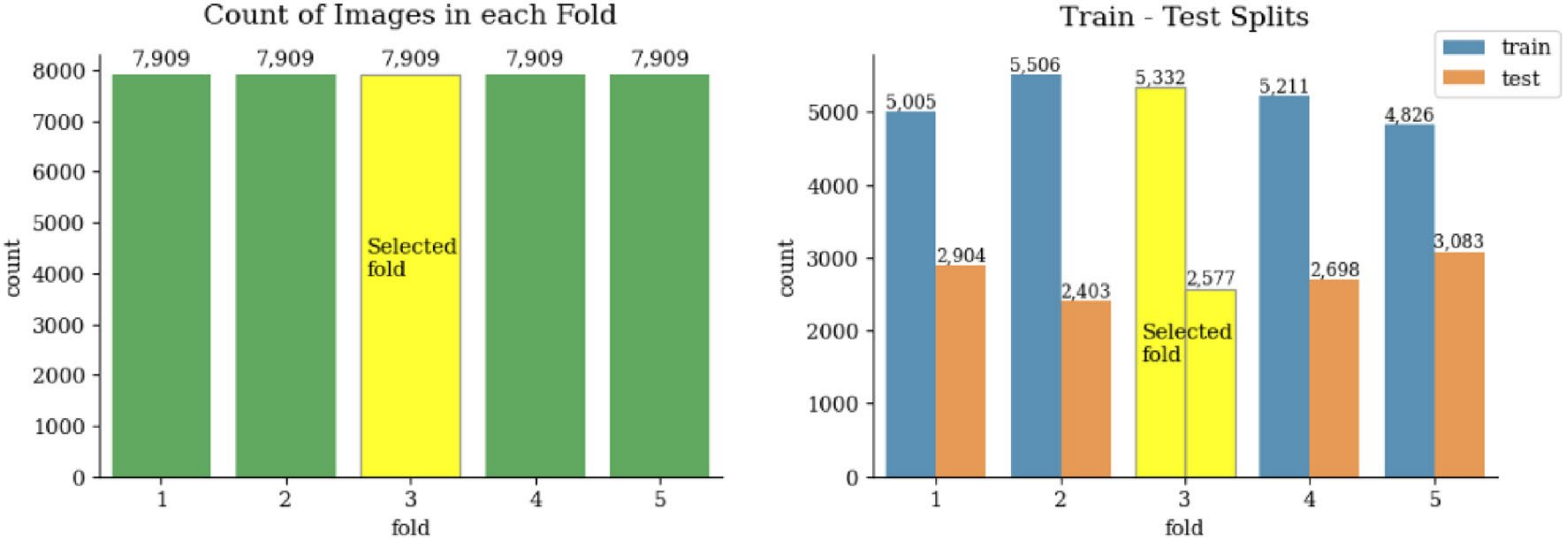
Five fold cross-validation strategy.

Exploratory Data Analysis (EDA) is an essential phase in the data analysis process that involves examining and understanding the underlying patterns, relationships, and characteristics of a dataset. It provides valuable insights into the data and helps in making informed decisions throughout the analysis. The dataset being used is partitioned into five subsets (fivefold cross), where each fold serves as both a training and testing set. The proposed approach used the third fold, since it has the highest proportion of training images to ensure that the training and testing data come from different individuals, reducing the risk of overfitting and providing a more accurate evaluation of the model’s generalization ability, as shown in Fig. 2.

#### Train set balancing

**Figure 3:**
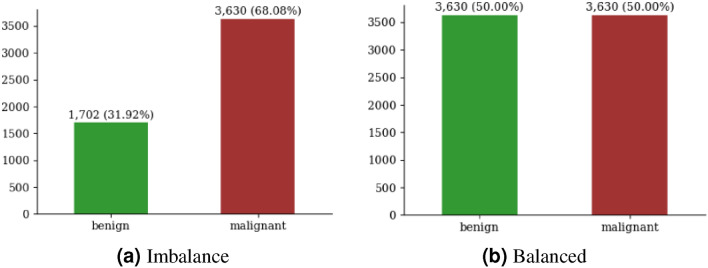
Imbalance and balanced dataset.

There is a clear difference between the classes within the selected third fold: malignant cases (3630) exceed benign cases (1702). As shown in Fig. 3(a), such imbalances can hinder accurate classification. Therefore, the up-sampling technique is employed to equalize the benign class with the malignant, as shown in Fig. 3(b). We especially use up-sampling in order to increase the number of instances in the minority class (benign class) and to balance the class distribution. We also used it because we didn’t want to discard potentially valuable data from the majority class (the malignant class).

### Data augmentation

**Figure 4:**
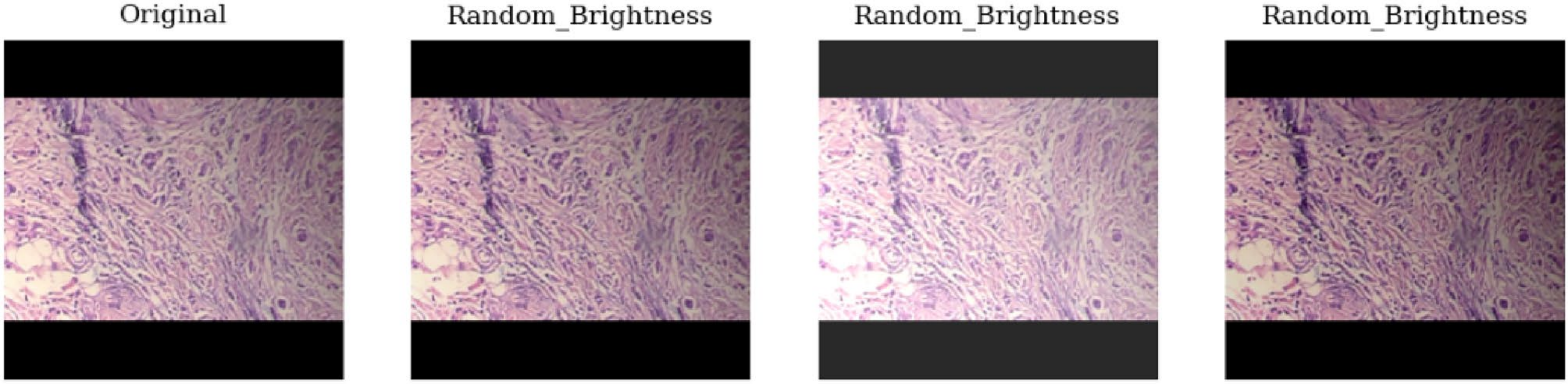
Random brightness of the original image in data augmentation.

**Figure 5:**
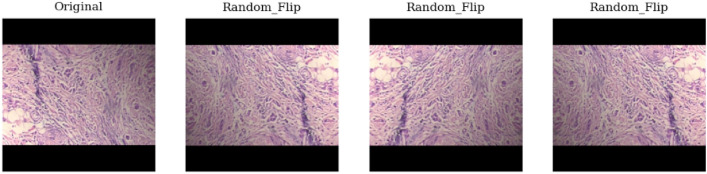
Random flipping of the original image in data augmentation.

**Figure 6:**
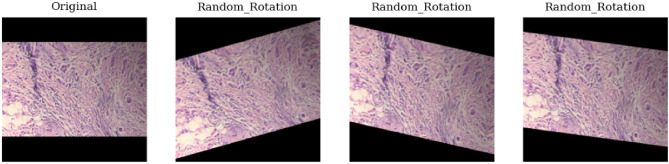
Random rotation of the original image in data augmentation.

Data augmentation serves as a strategic approach to counter overfitting^42^, enhance classification accuracy, and expand the sample size. The data augmentation used involves color transformations such as brightness, flipping and rotation , along with basic augmentation methods as shown in Figs. (4, 5 and 6). When rotating images randomly, zero-pixel margins are added to the edges. Image augmentation helps in increasing data and address overfitting in DCNN models.

### Feature extraction

CNNs excel at extracting crucial details from raw histopathological images; they can identify features without any prior knowledge. CNNs are great at identifying local features like nuclei, glands, and tissue boundaries. They’re also flexible enough to handle different sizes of features in the images. CNNs can adapt to scale variations present in histopathological images by using pooling layers and convolutional filters. The Custom CNN structure is divided into two main parts : feature extraction and classification. The convolutional layer initiates feature extraction, producing a “feature map” highlighting image aspects like corners and edges. As these features proceed through the network, deeper insights are gleaned. MobileNetV3-Small, tailored for mobile edge devices, integrates advanced mechanisms like bottleneck residual blocks and hard swish activation for resource efficient feature extraction. EfficientNetB0 employs strategies such as depthwise convolutions for efficient feature extraction without draining resources. The used VGG16 model combines convolutional layers with Max-Pooling and ReLU activation to extract and hierarchize features. Lastly, the used ResNet50V2 uses the initial layers and “residual blocks” for feature refinement, while skip connections ensure consistent information flow, streamlining feature extraction.

### Classification

**Figure 7:**
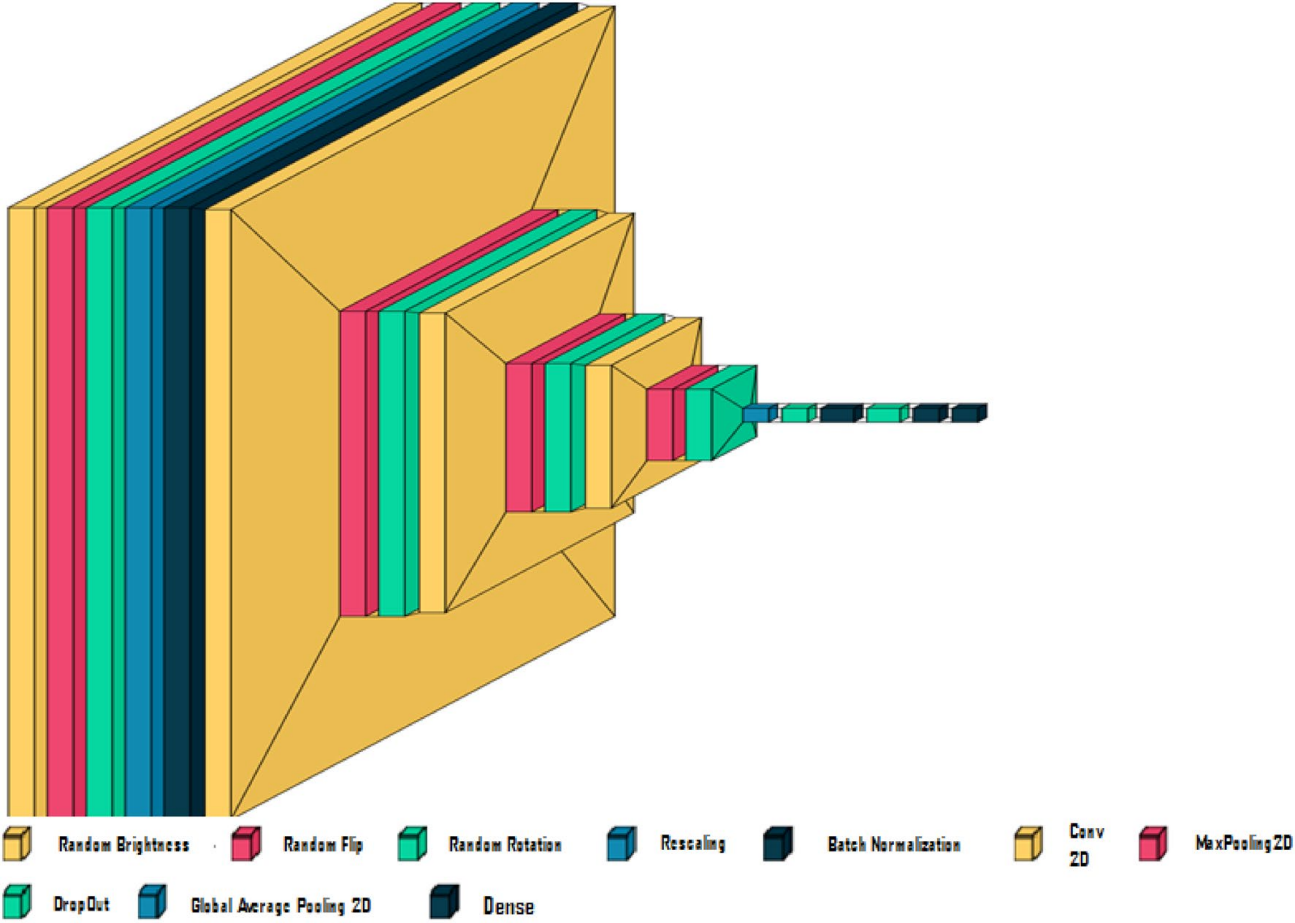
Custom CNN model.

The Custom CNN model shown in Fig. 7 is tailored for image classification, taking 224 $\times$ 224 pixel images with three color channels. It begins by augmenting data through methods like brightness adjustment and image flipping to improve the model’s resilience. The images are normalized between 0 and 1, and the inputs are standardized using batch normalization. The core of the model consists of three convolutional blocks, each with a 2D convolution, max-pooling, and dropout layers. A global average pooling layer reduces data size while maintaining information depth. The model concludes with three interconnected dense layers, using sigmoid and ReLU activation functions for classification and learning intricate patterns, respectively. It contains 108,603 parameters, of which 108,597 are trainable. Table 3 indicates that each pre trained model uses the Adam optimizer with a Learning Rate (LR) of 0.0005. The input image size was set to 224 $\times$ 224, and a kernel size of 3 was used. The model was trained with batch size 18 and for a total of 20 epochs to avoid overfitting of the training dataset, whereas too few may result in an underfit model. Early stopping is a method that allows you to specify an arbitrarily large number of training epochs and stop training once the model performance stops improving on the validation dataset. One of the best ways to choose the number of epochs is to experiment with different values and compare the results. You can start with a small number of epochs and gradually increase it until you see a significant improvement or a sign of overfitting.

The used MobileNetsV3 model demonstrates enhanced accuracy over its predecessor, the used MobileNetV2 model is attributed to the integration of the SE block and the h-swish activation function. the used EfficientNetB0 model excels in image classification and is preferred for transfer learning. However, its specific resolutions, not divisible by standard metrics, can lead to computational inefficiencies, notably in variants like B0 and B1 with resolutions of 224 and 240. Keras offers a spectrum of EfficientNet variants, B0 to B7, detailed in https://keras.io/examples/vision/image_classification_efficientnet_fine_tuning/ ResNet-50 is a CNN that is 50 layers deep. ResNet, short for Residual Networks is a classic NN used as a backbone for many computer vision tasks.Its architecture allows the training error to be reduced with a deeper network through connection skip.

**Table 3:** Values of the used parameters in the proposed approach.

Parameter	Value
Batch size	18
Optimizer	Adam
LR	0.0005
Size of image	224 $\times$ 224
Kernel size	3
Number of epoch	20

### Hyperparameter tuning

**Figure 8:**
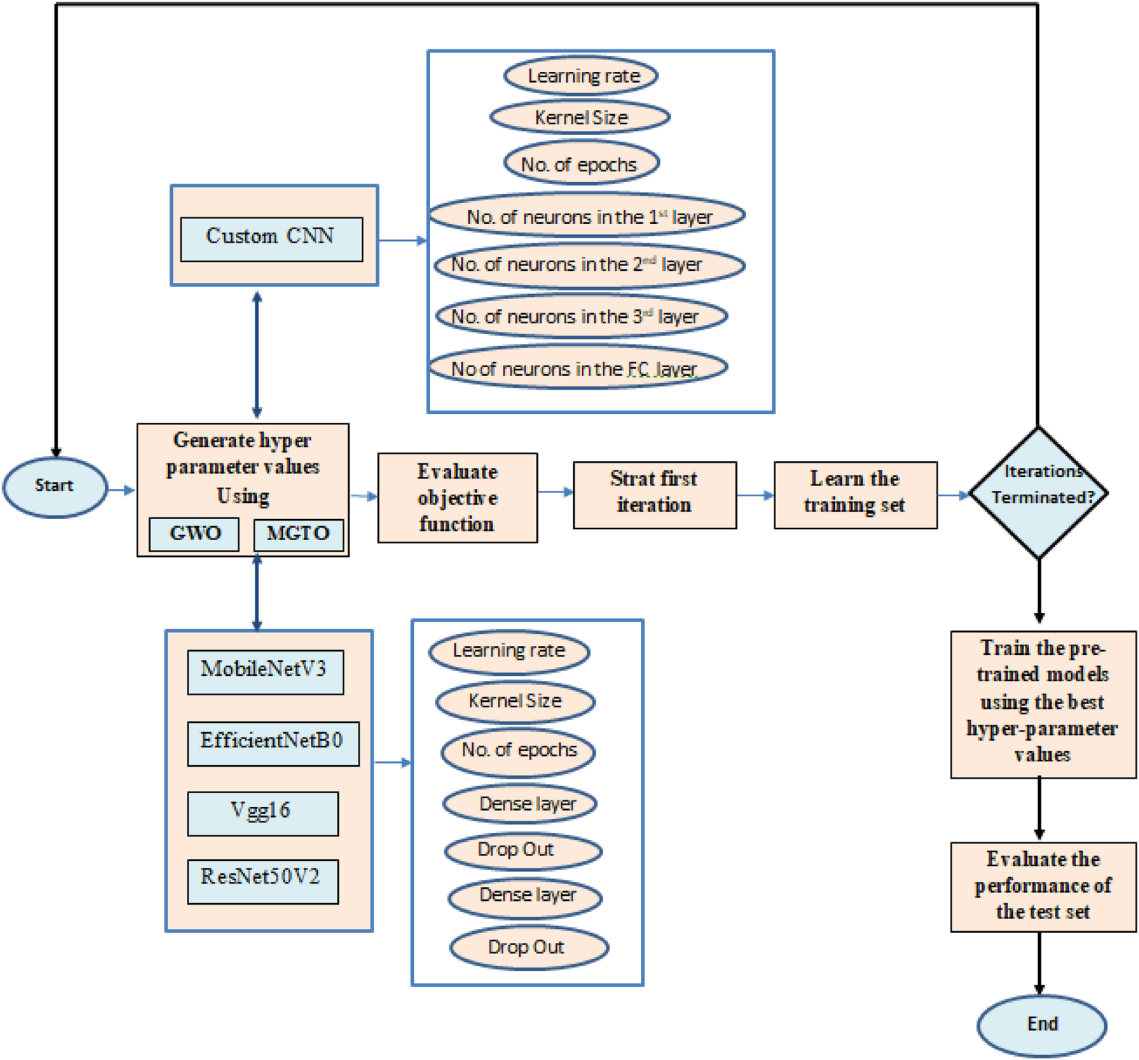
Fine tuning process used in the proposed approach.

The Custom CNN and the used pretrained models share common hyperparameters such as number of epochs, kernel size, and the LR of the Adam optimizer, they differ in several aspects. Specifically, the number of neurons in the three convolution layers and one Fully Connected (FC) layer of the Custom CNN are distinct from the pretrained models. Additionally, the pretrained models differ from the Custom CNN in terms of the dropout rate for both convolution and FC layers, as well as the number of neurons in these layers. The meta-heuristic algorithms, MGTO and GWO, are tasked with identifying optimal hyperparameter values. These algorithms begin with a random solution, described as an N-dimensional vector that corresponds to the hyperparameters of each model. Through iterative refinement, they aim to enhance this initial solution. Their optimization strategy relies on a fitness function, which assesses the performance of specific hyperparameter sets based on the five mentioned models. The goal is to fine-tune the hyperparameters to maximize the accuracy of the Custom CNN and the four other pretrained models, ultimately improving BT classification accuracy. For the Custom CNN, both GWO and MGTO metaheuristic optimizers are applied to tune its hyperparameters, as shown in Fig. 8. These optimizers also tune hyperparameters for models such as MobileNetV3, EfficientNetB0, Vgg16, and ResNet50V2. In separate processes, GWO and MGTO generate hyperparameter values and evaluate them using a fitness function to identify the best values. The process first checks the number of iterations; if they haven’t terminated, both optimizers generate new hyperparameters values and the training cycle is repeated; otherwise, the pretrained models undergo training with the best-identified hyperparameters. Finally, the models are tested with optimal hyperparameters to ensure both accuracy and efficiency.

**Algorithm 1:**
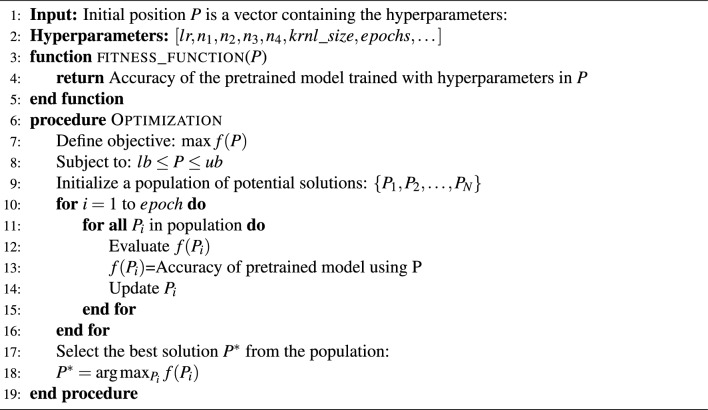
Fine tuning using MGTO.

Fitness function is used to measure how the current solution is close to the best solutions at every stage. This function mirrors the performance of the metaheuristic algorithms. Improving this function results in better accuracy for the five pretrained models used. In every step, the model operates with new hyper-parameters values. The fitness function value that calculated from classifying test data, is provided to the metaheuristic algorithms. This value serves as a cost function to adjust the succeeding hyper-parameter solution. As shown in Algorithm 1, the steps of using MGTO as a fine-tuning hyperparameter for pretrained models. It starts with defining the fitness function of a given position $P$ in the search space:

$$\begin{aligned} f(P) = \text {accuracy of the pretrained model trained with hyperparameters in } P, \end{aligned}$$

where $P$ is a vector containing the hyperparameters:($P = [lr, n_1, n_2, n_3, n_4, krnl\_size, epochs, \dots ]$), in which $lr$ represents the LR, $n_i$ is the number of neurons in the $i\text {th}$ layer, $i=1,2,3,4$, $krnl\_size$ represents the kernel size, and the $epochs$ are the number of epochs in each iteration, then defining the optimization problem that aimed to maximize the fitness function over the search space defined by the following bounds of each hyperparameter:

Objective: $$\max f(P)$$

Subject to: $$lb \le P \le ub$$

where lb and ub are vectors that contain the lower and upper bounds for each hyperparameter, respectively.

MGTO optimization is then used by initializing a population of potential solutions (hyperparameter sets) is initialized, and the size of this population is $N$ . $\{P_1, P_2, \dots , P_{N}\}$

and by evaluating the fitness of each potential solution $P_i$: $f(P_i)$

Then MGTO update the mechanism by updating each $P_i$ in the search for better solutions. The update mechanism is discussed in detail in MGTO^40^. This process is iterative and continues for a certain number of iterations (10).

Termination: After the predefined number of iterations (10), the best solution $P^*$ is selected as:

9 $$\begin{aligned} P^* = \arg \max _{P_i} f(P_i). \end{aligned}$$

Fine-tuning aims to identify the optimal set of hyperparameters $P^*$ that enhances the performance of the proposed method. This objective is achieved by emulating gorilla troop behaviors, facilitating the search, and updating of potential solutions across a defined number of iterations.

1. *The procedure:* The main procedure is fine tuning with GWO, it takes both train and test sets $train_ds$ (for training) and $test_ds$ (for evaluating model performance).
2. *Fitness function:* Inside the main procedure, there’s a function named *f* that evaluates how good a given set of model hyperparameters is. The hyperparameters for the model (like LR, number of neurons, kernel size, epochs, etc.) are packed into a list named ‘P‘. The used models are then trained using the training set to return the accuracy.
3. *Problem definition:* The goal of the optimization (what we want the GWO to solve) is defined in $\text {problem}_{\text {dict1}}$. This includes the fitness function, lower and upper bounds for the hyperparameters, and an objective (in this case, to maximize the accuracy).
4. *Grey wolf optimizer settings:* Some parameters for the GWO itself are set, like the number of iterations it will run ($\text {epoch}$) and the number of potential solutions it will consider ($\text {pop}_{\text {size}}$).
5. *Optimization:* An instance of the GWO algorithm is created and initialized with the above settings. The GWO then tries to solve the optimization problem, looking for the best set of hyperparameters for the used models.
6. *Results:* After all iterations of the GWO are complete, the algorithm extracts the best set of hyperparameters and the highest validation accuracy achieved. These results are then returned.

**Algorithm 2:**
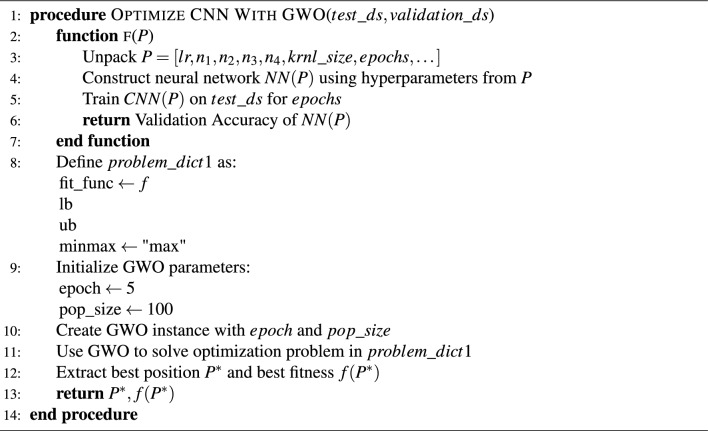
Fine tuning using GWO.

**Table 4:** Hyperparameters values in Custom CNN.

Hyper-parameters	Value
LR	[0.0001–0.0005]
Lower/Upper bound epochs	[5–30]
Number of neurons in the first layer	[16–32]
Number of neurons in the second layer	[32–64]
Number of neurons in the third layer	[64–128]
Number of neurons in the FC layer	[128–512]
Kernel size	[3–5]
Number of iterations	10

**Table 5:** Hyperparameters values in MobileNetV3, EfficientNetB0, Vgg16, and ResNet50V2.

Hyper-parameters	Value
LR	[0.0001–0.0005]
Lower/upper bound epochs	[5–30]
Drop-out	[0.2–0.5]
Dense	[128–256]
Drop-out	[0.2–0.5]
Dense	[32–64]
Kernel size	[3–5]
Number of iterations	10

Hyperparameters are trainable variables in a DL model that fall within specific ranges. Achieving desirable results from a DL mode require an optimal set of hyperparameter values. Fine-tuning these hyperparameters to obtain satisfactory outcomes is both laborious and precise. Gaining these desired outcomes often involves manually adjusting various hyperparameter combinations, relying on extensive experience, intuition, and deep knowledge of the model. Tables 4 and 5 provide a list of the most commonly used hyperparameters. In Table 4, LR [0.0001–0.0005] dictates how big of a step we take during optimization. A smaller LR ensures careful updates to avoid missing the best solution, while a larger one accelerates the process of finding a solution. We chose a range from 0.0001 to 0.0005 to find a good mix of accuracy and speed in reaching a solution. Epoch Range [5–30]: An epoch is one full cycle of passing the data through the network. By setting the range from 5 to 30 epochs, we’re testing different lengths of training to see how well the model learns over time without learning too much from the data (overfitting). Dropout [0.2–0.5]: Dropout helps in preventing the model from relying too much on certain patterns by randomly ignoring some neurons during training. By having dropout rates between 0.2 and 0.5, we ensure the model develops a well-rounded understanding by not depending too much on certain data points. Dense Layer Neurons [128–256 and 32–64]: These numbers indicate the neurons in the dense layers, which help the model understand complex patterns. We explore ranges from 128 to 256 and 32 to 64 neurons to balance the model’s complexity with its ability to process information efficiently. Kernel size [3–5]: Determines the size of the filter in convolutional layers, affecting what the model sees in the data. A range from 3 to 5 allows the model to recognize both small and large patterns, offering versatility in what it can learn from the data.

**Table 6:** Functions and impacts of various hyperparameters.

Hyperparameter	Effect	Impact of increase	Impact of decrease
LR of Adam optimizer	Determines the step size during training. Affects speed of weight updates.	Can cause unstable training or even divergence.	Can lead to slow convergence or getting stuck in local minimum.
Kernel size	Refers to filter size in conv layers. Impacts spatial field the network captures.	Captures larger spatial hierarchies, may lose fine details.	Can capture finer details, might miss broader patterns.
Number of epochs	Specifies number of times dataset is seen during training.	Can lead to better training accuracy but might overfit.	Might underfit if too low.
Neurons in convolution and FC layers	Determines model’s capacity or complexity.	Increases model capacity but may overfit.	Might be too simple to capture complex patterns.
Dropout rate	Regularizes the model by setting fraction of units to 0 during training.	More regularization, might reduce overfitting but can lead to underfitting if too high.	Less regularization, risk of overfitting.

The function and the effect of increasing and decreasing the used hyperparameter are listed in Table 6. The proposed approach streamlines the hyperparameter tuning process. GWO and MGTO employ these hyperparameters during model training, while selected evaluation metrics assess the model’s performance in each trial. This iterative procedure continues either until the termination criteria are met or after 10 iterations are completed.

## Experimentation and results

### Environment setup

All experiments in this work are conducted on a PC with the following properties: Windows 7 with an Intel(R) Core(TM) i7-3687U CPU @ 2.10 GHz 2.60 GHz and 8.00 GB of RAM.

### Measurement and performance evaluation methods

#### Confusion matrix

**Figure 9:**
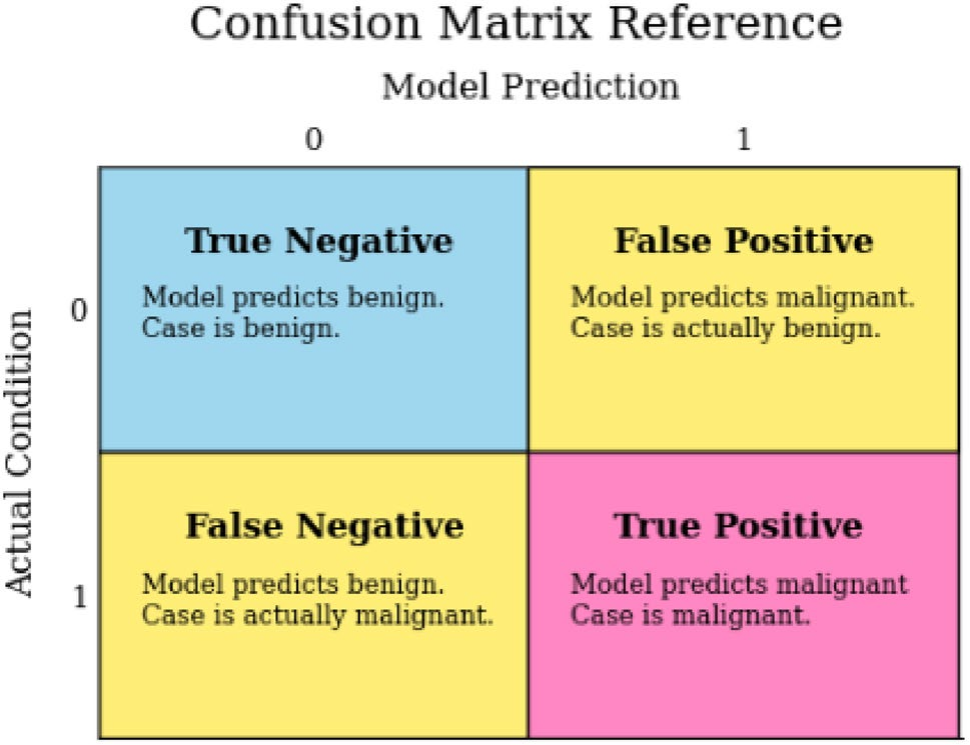
Confusion matrix.

A confusion matrix serves to measure a classification algorithm’s performance by identifying the correct and mistaken categorizations. This matrix consists of two axes : actual and forecasted values, depicted in Fig. 9. “Actually” denotes the precise classifications, whereas “predicted” represents the algorithm’s estimations. The values within each cell of the matrix, show the frequency of that particular pairing.

(1) True Positive (TP) represents the instances classified as BC. (2) True Negative (TN) refers to healthy subjects correctly classified as healthy. (3) False Positive (FP) denotes healthy subjects misclassified as BC. (4) False Negative (FN) indicates BC cases misclassified as healthy.

The evaluation metrics are employed to assess the performance of classifiers. In this paper, different performances evaluation metrics are utilized, expressed in Eqs. (10) to (13).

**Classification accuracy:** reflects the overall performance of the classification system, representing the diagnostic test’s probability of correct results is presented as follows:

10 $$\begin{aligned} \text {Accuracy} = \frac{(TN + TP)}{(TP + TN + FP + FN)} \times 100. \end{aligned}$$

Precision or specificity quantifies the accuracy of the model’s predictions; this metric measures the proportion of true positive predictions made by the model, compared to the total number of positive predictions. It is useful for tasks where false positives are particularly costly or harmful. it is calculated as:

11 $$\begin{aligned} \text {Precision} = \frac{TP}{TP + FP}. \end{aligned}$$

On the other hand, recall, or sensitivity, assesses the model’s ability to correctly predict the actual target values. This metric measures the proportion of true positive predictions made by the model, compared to the total number of actual positive cases. It is useful for tasks where false negatives are particularly costly or harmful, computed as:

12 $$\begin{aligned} \text {Recall} = \frac{TP}{TP + FN}. \end{aligned}$$

The previous two metrics are subsequently utilized to determine the F1-Score, which provides a comprehensive measure of the model’s efficacy in discerning and forecasting target values from a specified dataset. This metric is a combination of precision and recall. It is calculated as the harmonic mean of precision and recall; the F1-Score value is computed as:

13 $$\begin{aligned} F1\text {-Score} = \frac{2 \times (\text {Precision} \times \text {Recall})}{\text {Precision} + \text {Recall}}. \end{aligned}$$

#### ROC-AUC

**Figure 10:**
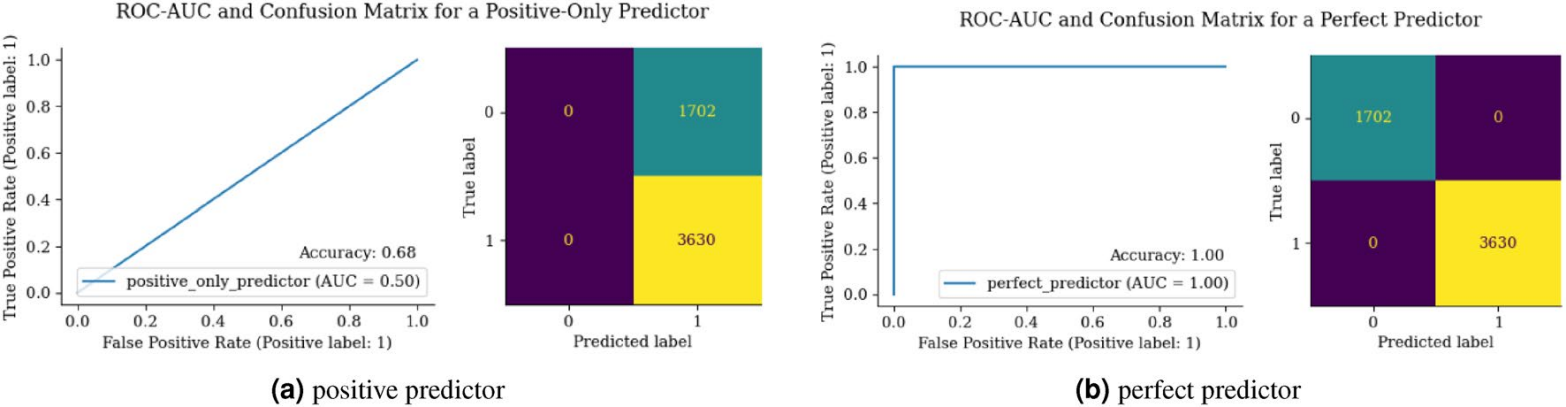
ROC-AUC for positive and perfect predictor.

ROC is a graphical representation used to assess the performance of a classification model across different threshold levels as shown in Fig. 10(a, b). It plots the TP Rate (TPR) against the FP Rate (FPR), providing insights into the model’s performance in various classification scenarios. It is used for visualizing the trade-off between correctly identifying positive cases (TPR) and incorrectly labeling negative cases as positive (FPR).

#### Loss function

There are different kinds of loss functions used in DL. A binary cross-entropy loss function is used for binary classification tasks, it measures the dissimilarity between predicted and target probability distributions. https://medium.com/@amanatulla1606/demystifying-loss-functions-in-deep-learning-understanding-the-key-metrics-for-model-optimization-a81ce65e7315 (loss functions in DL).

### Results and discussions

Although the Custom CNN model surpasses the performance of other pretrained models, as presented in Table 7, its accuracy remains relatively low. Therefore, to enhance this accuracy, it becomes necessary to implement meta-heuristic optimizers on each pretrained model as fine-tuning.

**Table 7:** ROC-AUC, Acc, and Loss of all pretrained models in the proposed approach.

Fold	Pre-trained model	ROC-AUC	Accuracy	Loss	Augmentation	Time per each epoch (s)
Fold 3	Custom CNN	0.92259	0.84362	0.38658	Random brightness (0.2) Random Rotation (0.2) Random Flip	32
	MobileNetV3	0.80057	0.74699	0.52185		24
	Efficient Net	0.86750	0.82266	0.41202		30
	Vgg16	0.89827	0.78735	0.51558		51
	ResNet50V2	0.86441	0.77998	0.45359		34

**Figure 11:**
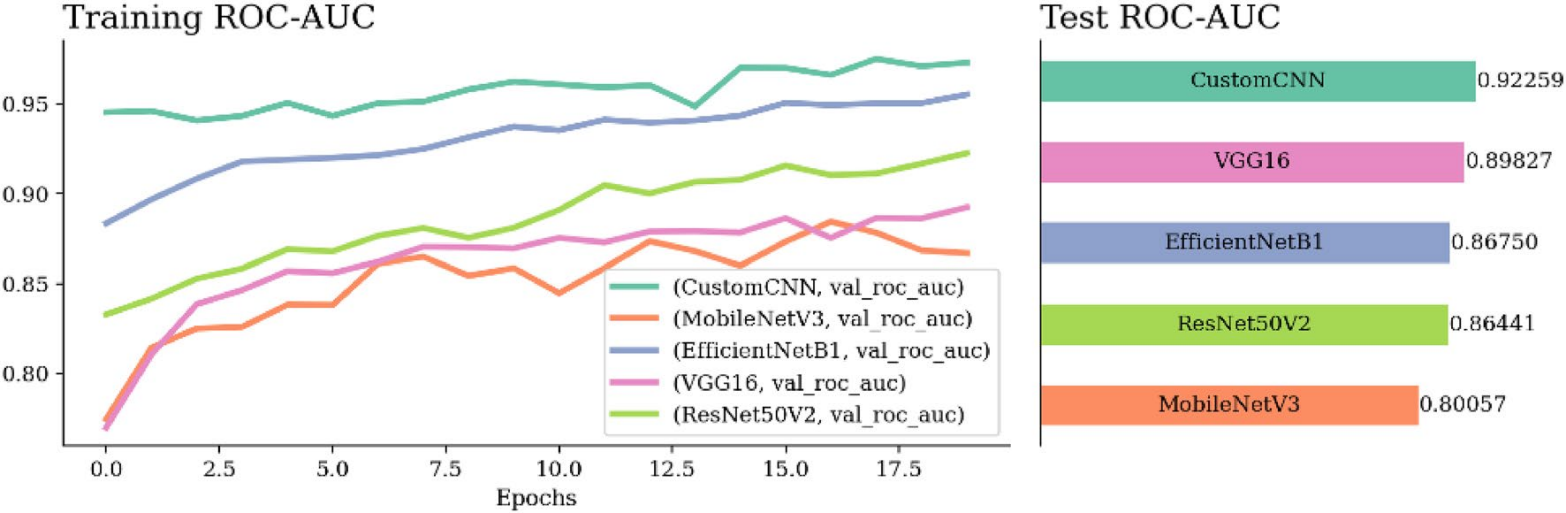
Training ROC-AUC of the used five different models in the proposed approach.

Figure 11 shows that Custom CNN demonstrates superior ROC-AUC over the four used models, achieving a score of 92%. On the other hand, VGG16, EfficientNetB0, ResNet50V2, and MobileNetV3 achieved ROC-AUC scores of 89%, 87%, 86%, and 80%, respectively. These results indicate that the Custom CNN model outperforms the other models for the given task.

**Figure 12:**
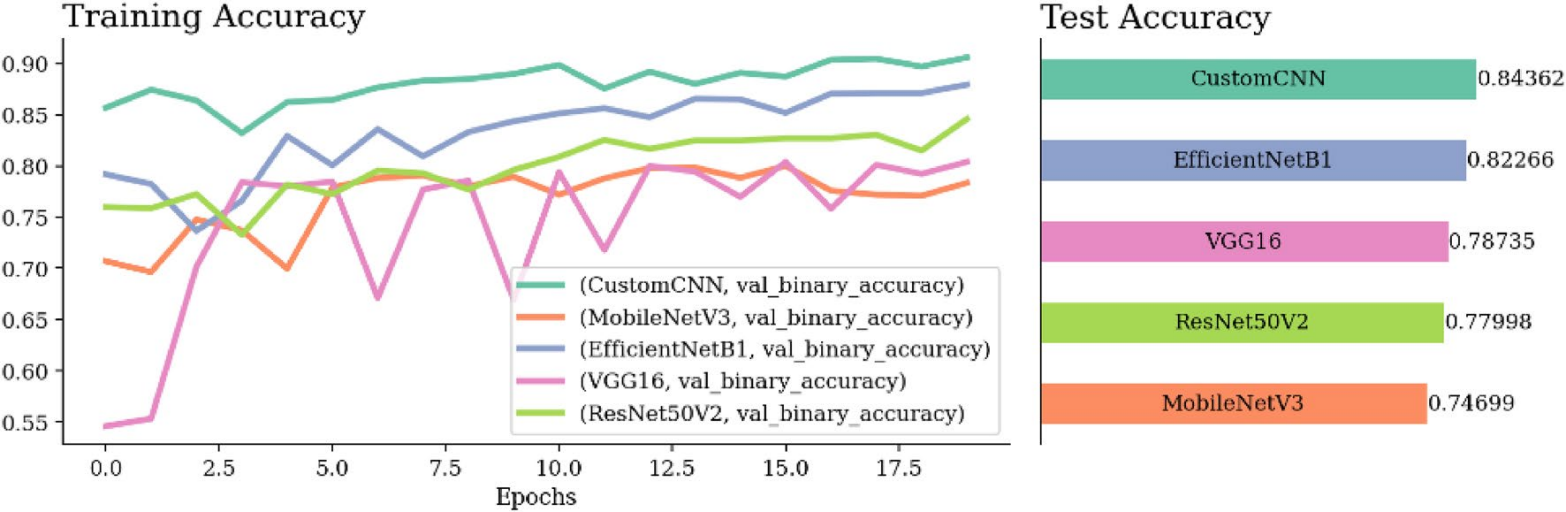
Training accuracy of the used five different models in the proposed approach.

Figure 12 provides a comparative analysis of accuracy results for the five models within the primary method. Evidently, the Custom CNN model provides exceptional performance by achieving an accuracy rate of 84%, while the other models achieve at most 78%.

**Figure 13:**
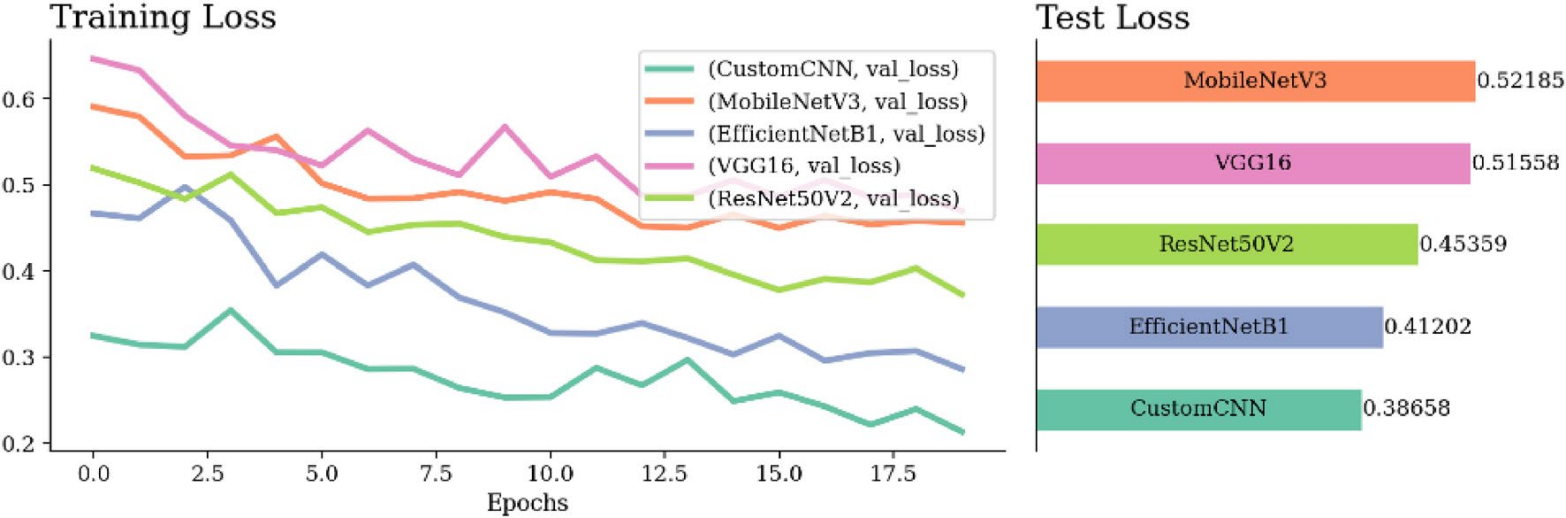
Training loss of the used five different models in the proposed approach.

Figure 13 presents a comparative analysis of loss results for five models within the primary method. Custom CNN model provides exceptional performance by achieving the lowest loss rate of 38%, while the other models achieve at least 41%.

**Figure 14:**
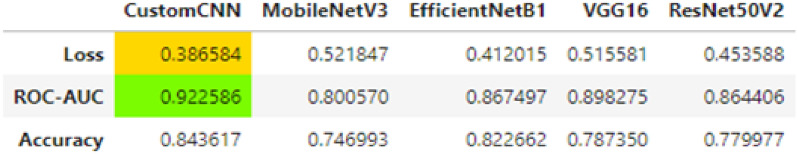
Comparison results of the used pretrained models in the proposed approach.

Figure 14 shows a comparative analysis of Custom CNN and the four pretrained models, presenting their respective loss, ROC-AUC, and accuracy metrics. Custom CNN outperforms the other models in performance, achieving the highest ROC-AUC at 0.92 and the highest accuracy at 0.84. Additionally, it achieves the lowest loss value at 0.38.

**Figure 15:**
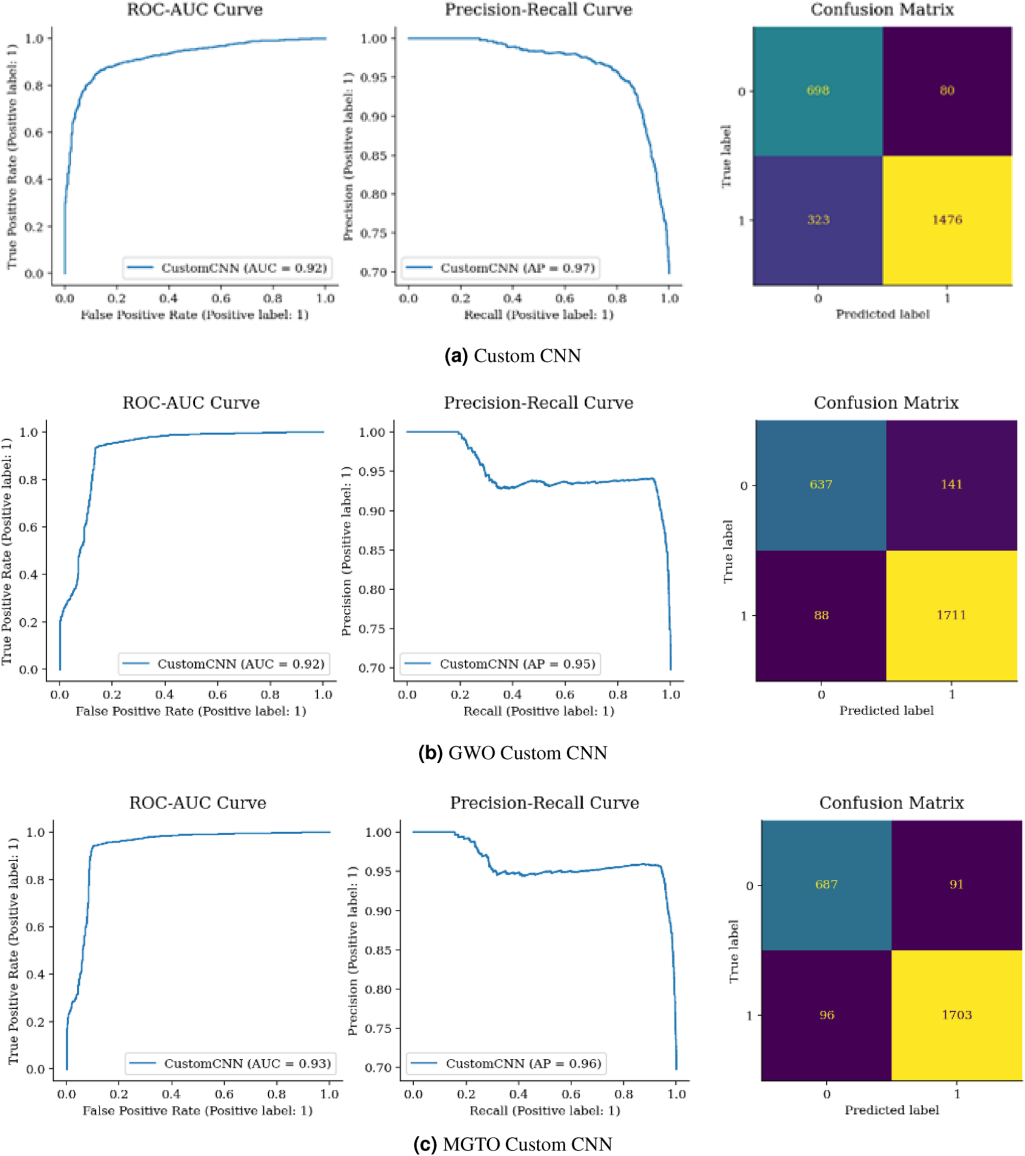
Confusion matrix and ROC-AUC of Custom CNN, GWO Custom CNN, and MGTO Custom CNN.

Figure 15(a) shows that the benign samples were misclassified as malignant FP and 323 malignant samples inaccurately classified as benign FN. Conversely, the model correctly identified 1476 malignant cases TP, and 698 benign cases TN. Figure 15(a–c) shows that GWO Custom CNN has the most TP compared to TN in MGTO Custom CNN by 50 samples. Additionally, GWO Custom CNN has FP and FN (229), higher than MGTO Custom CNN (187). Consequently, MGTO Custom CNN outperforms both GWO Custom CNN and Custom CNN in terms of overall accurate classifications.

**Figure 16:**
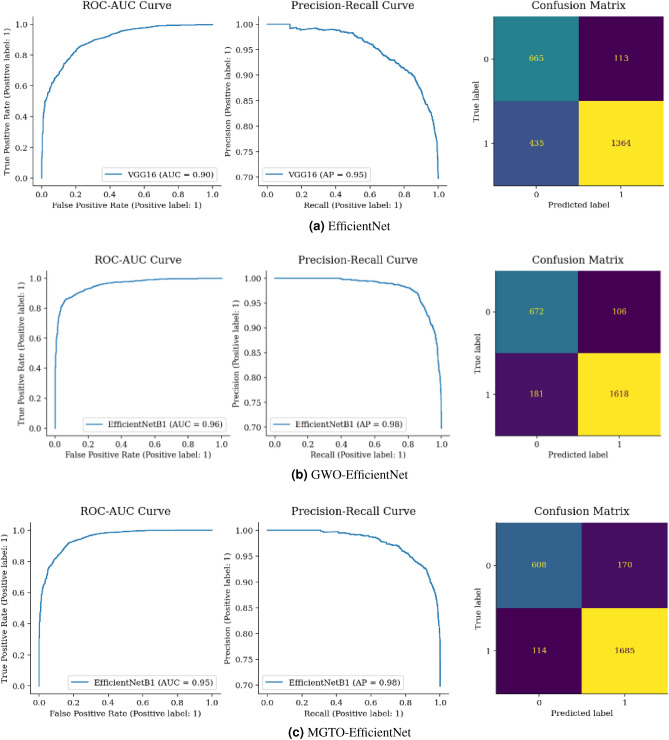
Confusion matrix and ROC-AUC of EfficientNet, GWO-EfficientNet, and MGTO-EfficientNet.

Figure 16 shows 113 FP, where benign was misclassified as malignant and 435 FN where malignant samples were mistaken as benign. However, the model correctly identified 1364 malignant TP and 665 benign TN cases. Comparatively, while GWO EfficientNet has a slightly higher TN of 672, MGTO EfficientNet excelled with a TP of 1685, surpassing GWO’s 1618. In general, MGTO EfficientNet has more correct classifications at 2293 versus GWO’s 2290 and fewer mistakes (284 compared to GWO’s 287), as detailed in Fig. 16(a–c).

**Figure 17:**
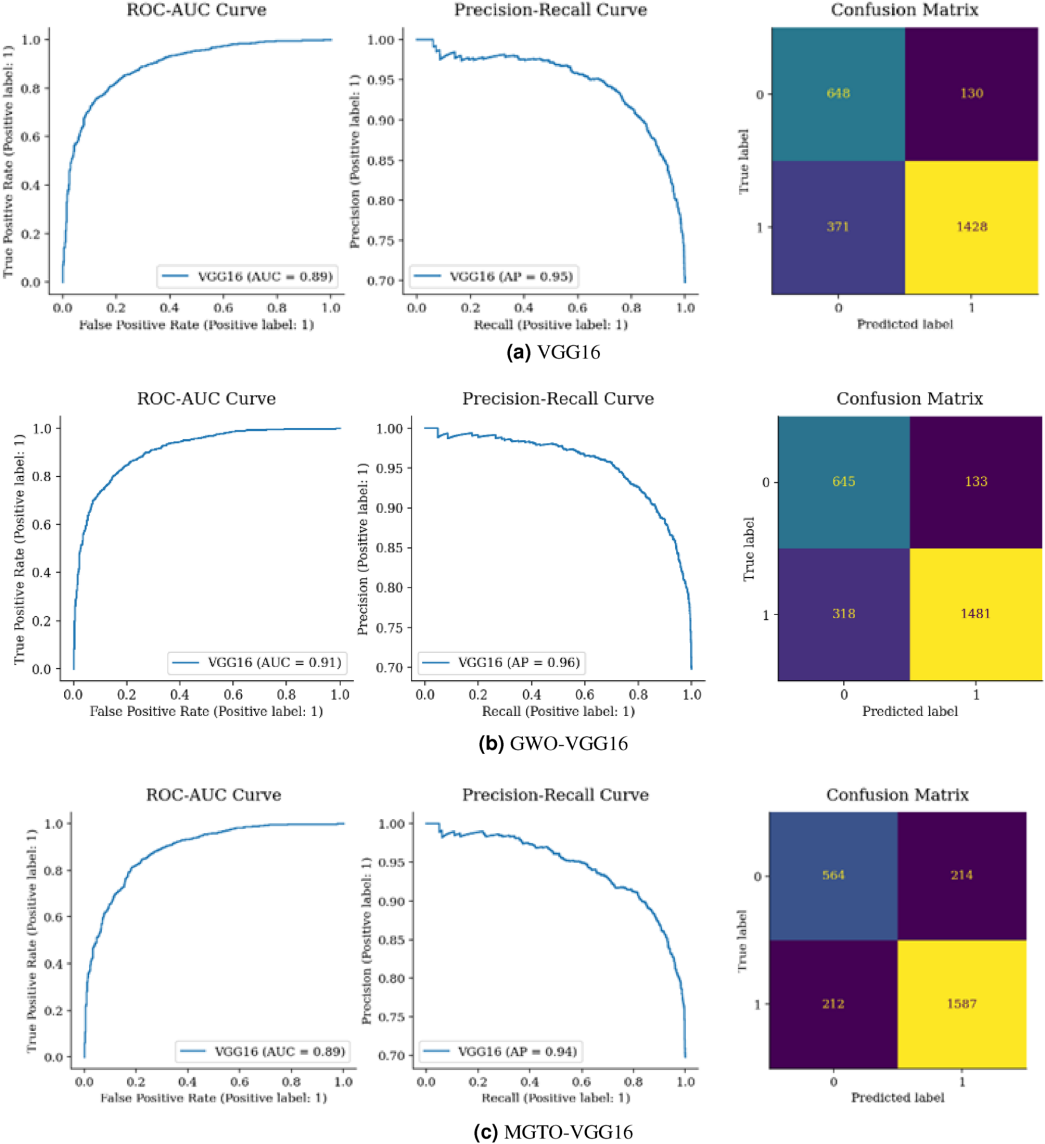
Confusion matrix and ROC-AUC of VGG16, GWO-VGG16, and MGTO-VGG16.

Figure 17(a) shows that 1428 malignant and 648 incorrectly classified cases are present when Vgg16’s is used. However, there were 501 incorrect classifications (130 + 371). When comparing the total correct classifications, Vgg16 had 2076, while GWO Vgg16 and MGTO Vgg16 had 2126 and 2151, respectively, as shown in Fig. 17(a–c). Despite the minimal increase in correct classifications, MGTO Vgg16 outperformed both GWO Vgg16 and Vgg16.

**Figure 18:**
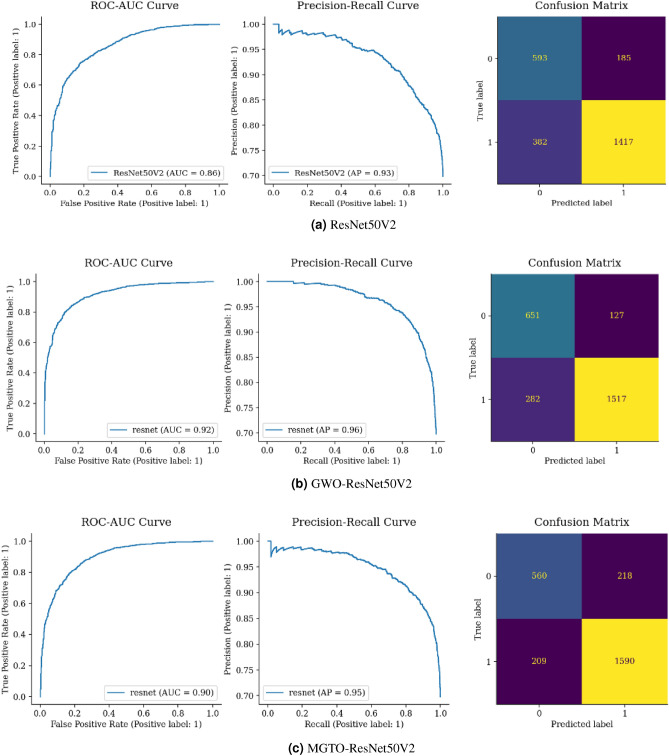
Confusion matrix and ROC AUC of ResNet50V2, GWO-ResNet50V2, and MGTO-ResNet50V2.

Figure 18(a) displays a confusion matrix for ResNet, correctly classifying 2010 instances. Yet, GWO ResNet and MGTO ResNet in Fig. 18(b,c) outperformed with 2168 and 2150 correct classifications, respectively, with GWO ResNet is the most accurate.

**Figure 19:**
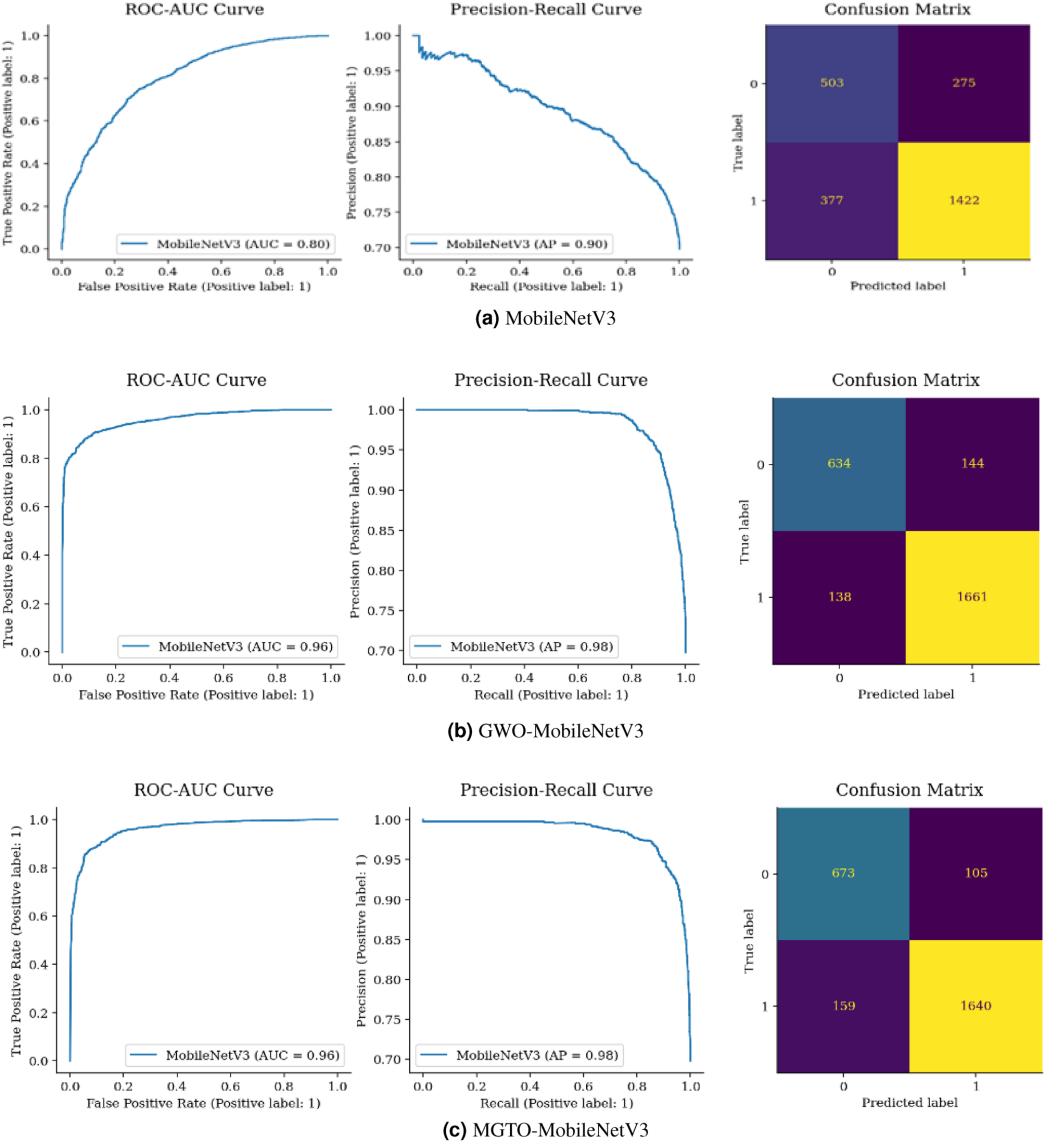
Confusion matrix and ROC-AUC of MobileNetV3, GWO-MobileNetV3 and, MGTO-MobileNetV3.

Figure 19(a) illustrates 275 benign samples misclassified as malignant FP and 377 cancerous samples inaccurately classified as normal FN. The model correctly identified 1422 malignant cases TP and 503 benign ones TN. Upon comparing Fig. 19(a–c), MGTO-MobileNet outperformed both GWO-MobileNet and MobileNet. Moreover, MGTO-MobileNet’s (264) has FP and FN (282) lower than GWO-MobileNet’s. Therefore, it is the superior model in terms of accuracy compared to its counterparts.

From Figs. 15, 16, 17, 18 and 19, it is clear that the MGTO optimizer achieves higher accuracy over the GWO optimizer in Custom CNN and Vgg16 within 10 iterations only. All conducted experiments were running on an Intel(R), Core(TM), i7-3687U CPU @ 2.10 GHz 2.60 GHz and 8.00 GB of RAM, the overall performance may be changing to be higher if high-performance computing is used.

As presented in Tables 8 and 9, the best values of hyperparameters for each of the pretrained models and custom CNN was generated by both MGTO and GWO after 10 iterations. The hyperparameters used in Table 8 are different from that of Table 9 and that is due to the architecture of each pretrained model. As presented in Table 8, the comparison between the Custom CNN models optimized using MGTO and GWO reveals that MGTO CNN outperforms GWO CNN in terms of accuracy and ROC AUC. This superior performance can be attributed to several factors, including MGTO’s possibly more effective hyper-parameter search strategy, the specific combination of hyperparameters it identified (such as LR and filter counts in the network layers), and the longer training duration (25 epochs versus 11). Moreover, the inherent randomness in optimization processes might have favored MGTO for this specific run.

**Table 8:** Hyper parameters of both GWO and MGTO within 10 iterations in the Custom CNN.

Optimizer	Model	New paramters within 10 iterations							Metrics		
		Adam LR	No of neurons in 1st layer	No of neurons in 2nd layer	No of neurons in 3rd layer	No of neurons in FC layer	Kernel Size	Epochs	ROC ACU	Acc	Loss
GWO	Custom CNN	0.00014	17	52	121	449	4	11	0.91	0.91	0.41
MGTO	Custom CNN	0.00019	18	35	121	483	3	25	0.95474	0.9313	0.40

**Table 9:** Hyper parameters of both GWO and MGTO within 10 iterations in the four used pretrained models.

Optimizer	Model	New parameters within 10 iterations							Metrics		
		Adam LR	Drop Out	Dense layer	Drop out	Dense layer	Kernel size	Epochs	ROC ACU	Acc	Loss
GWO	Mobile Net	0.00035	0.2410	0.2308	181	35	4	27	0.92	0.89	0.59
	Efficient Net	0.00048	0.3074	146	0.3426	37	3	17	0.95	0.88	0.32
	VGG 16	0.00011	0.4432	231	0.3062	31	3	27	0.91	0.79	0.57
	Res Net	0.00031	0.8610	401	0.8848	25	4	22	0.91	0.84	0.61
MGTO	Mobile Net	0.00035	0.3358	161	0.3812	56	4	22	0.94	0.89	0.62
	Efficient Net	0.00038	0.4956	152	0.4528	50	3	10	0.93	0.88	0.42
	VGG 16	0.00048	0.3716	246	0.3426	26	3	17	0.90	0.82	0.45
	Res Net	0.00022	0.26433	375	0.5168	28	4	24	0.89	0.83	0.37

Table 9 delineates the performance metrics and hyperparameters of pretrained models that have been fine-tuned using GWO and MGTO. Each pretarined model is benchmarked on metrics like ROC-AUC, accuracy, and loss over ten iterations. EfficientNetB0 consistently demonstrates strong performance under both optimizers with GWO yielding a slightly higher ROC-AUC of 0.95 compared to MGTO’s 0.93. However, the accuracy of EfficientNet remain consistent at 0.88 for both optimizers. MGTO VGG 16 boasts a slightly higher accuracy (0.82) than its GWO counterpart (0.79). Conversely, GWO’s ResNet garners a modestly superior accuracy of 0.84 compared to MGTO’s ResNet is at 0.83. The minor discrepancies in accuracy between MGTO’s VGG 16 and GWO’s VGG 16 might be attributed to MGTO’s hyperparameters for VGG 16 being marginally better optimized for the specific dataset. This could include factors like dropout rates, which influence model generalization, or even the number of dense layers, which impact model depth and capacity.

For ResNet, GWO’s slightly better performance could stem from the particular interplay of the model’s depth and the optimization strategy of GWO, suggesting that for certain architectures, one optimization method might offer subtle advantages over the other.

**Table 10:** Evaluation metrics of each model without optimization and with two different optimizers.

Model	Basic	GWO	MGTO	Basic	GWO	MGTO	Basic	GWO	MGTO
	ROC-AUC			Accuracy			Loss		
Custom CNN	0.92259	0.91	0.95474	0.84362	0.91	0.93	0.38658	0.41	0.40
MobileNetV3	0.80057	0.92	0.94	0.74699	0.89	0.89	0.52185	0.59	0.62
Efficient Net	0.86750	0.95	0.93	0.82266	0.88	0.88	0.41202	0.32	0.42
Vgg16	0.89827	0.91	0.90	0.78735	0.79	0.82	0.51558	0.57	0.45
ResNet50V2	0.86441	0.91	0.89	0.77998	0.84	0.83	0.45359	0.61	0.37

The equivalent accuracies observed for both MGTO and GWO in the case of MobileNet and EfficientNet imply that these models might have reached a performance plateau for the specific task, and there is no optimization strategy offers a distinct edge over the other. Fine-tuning models using different optimizers can yield varying results based on the synergies between the model’s architecture and the optimizer’s strategy. In this context, while MGTO offers slight advantages for VGG 16, GWO emerges as marginally superior for ResNet. Meanwhile, for both MobileNet and EfficientNet, the choice of optimizer doesn’t seem to significantly alter performance. Optimization techniques enhance performance across various models, notably improving Custom CNN. Other models, like MobileNetV3, Vgg16, and ResNet50V2 also benefit, with Efficient Net showing the most significant improvement, particularly with MGTO optimization. MGTO consistently outperforms GWO, notably in Efficient Net and Custom CNN. Overall, optimization techniques, especially MGTO, enhance model performance across different types, as illustrated in Table 10. As indicated in Table 11, the proposed approach, MGTO Custom CNN, outperforms the existing state-of-the-art methodologies, even though the MGTO optimizer uses only ten iterations. The model doesn’t restrict itself to specific magnifications for training and testing but operates across different magnifications. Other models, in contrast, work with each magnification separately, measure the accuracy, and then compute VGG16 and ResNet primarily as feature selectors and other algorithms for classification. On the other hand, the proposed approach uses them, along with others, as pre-trained models capable of both feature extraction and selection from the image, as well as the subsequent classification.

**Table 11:** Comparison with the state-of-art methods.

Reference/year	Dataset	Name of optimizer	Hyper-parameters	Results
^19^/2021	BreakHis dataset	BiT Hyper Rule for fine-tuning parameters in Big Transfer.	Resizing: (224 $\times$ 224), (331 $\times$ 331) Rescaling: (0–1)	Accuracy: 90% Benign: Precision: 0.29 Recall: 0.27 F1-Score: 0.28 Malignant: Precision: 0.66 Recall: 0.68 F1-Score: 0.67
^32^/2021	Publicly available dataset based on histopathology images	Fine-tuning in a supervised fashion after pre-training in an unsupervised manner.	LR: 0.0001. Momentum: 0.9 Epochs for RBM training: 30 Weight decay: 0.00001 Patch size: 32 x 32. Epochs for fine tuning: 3000	Accuracy: 86% Sensitivity: 87.9% Error Rate:14% False Positive Rate: 15.9% Specificity:84%
^26^/2023	Breast Ultrasound Images (BUSI) dataset	A logistic regression classifier as a meta-learner to make our final prediction.	Max epochs 30 batch 32 Optimizer Adam Loss function Binary cross-entropy LR 0.0001 Range of rotation Random(0.5) Shuffling Yes Flip Neares	Accuracy: 90% Ensemble Meta-Model Benign Precision: 0.86 Recall: 0.95 F1-Score: 0.90 Malignant Precision: 0.94 Precision: 0.84 Recall: 0.89
^27^/2023	BreakHis dataset	Layer-wise fine-tuning, specifically using the AlexNet network	LR Momentum Scheduling rate Mini-batch size Epochs per layer	Acc of (40$\times$) = 93.6% Acc of (100$\times$)=91.3% Acc of (200$\times$) = 93.8% Acc of (400$\times$) = 89.1% The average = 91.925%
^29^/2020	BreakHis dataset	Layer-wise fine-tuning.	LR (0.001) Momentum (0.9) Scheduling rate (0.95) Mini-batch size (32) Epochs per layer (50).	Acc: 89.31% for 40$\times$ Acc: 85.75% for 100$\times$ Acc: 83.95% for 200$\times$ and Acc: 84.33% for 400$\times$.
^30^/2022	BreakHis BraTS NIH-Xray	Adaptive Hyperparameter Tuning (AHT)	Momentum (0.9)	Acc for BraTS = 91.08% Acc for BreakHis=91.26% Acc for NIH X-ray = 93.21%
^28^/2023	MIAS INbreast WDBC	Particle Swarm Optimization (PSO) Dragon-Fly Optimization Algorithm (DFOA) Crow-Search Optimization Algorithm (CSOA)	Scheduling rate (0.95)	Initial results Acc with wKNN, GNB, LSVM: 59.13–75.65% for MIAS 58.41–74.34% for INbreast 93.15–95.67% for WDBC datasets After applying the transformation techniques: CSOA-wKNN gave Acc of 84.35% for MIAS, 83.19% for INbreast, and 97.36% for WDBC datasets.
^18^/2022	BCDR Mammogram images	Transfer Learning (TL) using models like ResNet18	Mini-batch size (32)	Before TL: Classical ResNet accuracy is 67% After TL: Classical ResNet accuracy is 84%
Proposed approach (MGTO Custom CNN)	BreakHis dataset	MGTO	Kernel size Epochs, LR Numbers of filters in the 1st layer Numbers of filters in the 2nd layer Numbers of filters in the 3rd layer Numbers of filters in the FC layer	Accuracy: 93.13% For benign: (Precision) 1.00 Recall: 0.86 F1-Score: 0.92 For malignant: (Precision) 0.95 Recall: 1.00 F1-Score: 0.89

## Conclusion and future works

This paper has presented a proposed approach for the classification of BTs using a Custom CNN four other pretrained models. The initial findings were promising, with the Custom CNN model achieving an accuracy of 84%. The application of optimization techniques, namely GWO and MGTO, is applied to each model. There was a marked improvement in performance, with the MGTO-optimized Custom CNN model achieving a remarkable 93.13% accuracy in just 10 iterations. This performance not only surpasses other state-of-the-art methods but also underscores the efficacy and significance of these optimization strategies in improving diagnostic tools for breast tumors, as demonstrated through experiments on the BreakHis dataset.

The mentioned models are experimented with a higher number of epochs, specifically setting it to 50, surpassing the previously mentioned 20 epochs. The outcome of this extended training duration resulted in the activation of Early stopping. This technique permits the specification of a significantly large number of training epochs, stopping the training process once the model’s performance ceases to show improvement on the validation dataset.

Despite these exciting findings, there are a few key points we need to keep in mind for future work. First, we haven’t yet tested our model in a real-time environment to see how well it works in the actual BT classification. This is an important step we’re missing. Second, our method needs a lot of computer power for training and making it better, which might be hard to find in places with limited resources. This could make it tough to use our method everywhere it’s needed. Moreover, while our model demonstrates superior performance, it is important to consider the context of these achievements. Some literature, such as the work by Joseph et al.^16^, reports higher performance metrics; however, these results may be subject to overfitting, casting doubt on their generalizability. Similarly, other studies, like that of Ijaz et al.^25^, have applied their models at only one magnification level, which may limit the applicability of their findings across varying conditions. These observations underscore the importance of cautious interpretation of comparative performance metrics and highlight the need for comprehensive testing across diverse conditions to ensure robust and reliable model performance. Despite these issues, our work adds valuable information on how to better classify BTs. Our success with optimization strategies, especially, points to new ways to improve diagnostic tools. But there’s more to do. We need to look at other ways to make DCNN models even better, maybe by preparing the data in new ways. Using the BreakHis dataset was a good start, but using more and different kinds of data in the future will make our model even stronger. Trying out other optimization methods could also give us better results. Lastly, if we can put these improvements into real-world BC detection systems, we could help catch the disease earlier and improve treatment and quality of life for many people.

## Data Availability

The used dataset (BreakHis) is publicly available from URL https://www.kaggle.com/datasets/ambarish/breakhis.
